# FLASH radiotherapy and immunotherapy synergy: mechanisms, strategies, and clinical translation prospects

**DOI:** 10.3389/fimmu.2026.1788149

**Published:** 2026-03-19

**Authors:** Qianyi Liu, Xianhu Zeng, Yongze He, Linsen Zhou, Ying Tang, Shuotong Liu, Xiaobin Wang, Shiyan Shen, Jialin Ji, Zhen Liu, Jiangping Li

**Affiliations:** 1 West China School of Medicine, Sichuan University, Chengdu, Sichuan, China; 2 Department of Radiotherapy Physics & Technology, Cancer Center, West China Hospital, Sichuan University, Chengdu, Sichuan, China; 3 Division of Thoracic Tumor Multimodality Treatment, Cancer Center, West China Hospital, Sichuan University, Chengdu, Sichuan, China

**Keywords:** cancer immunology, FLASH-RT, immunotherapy synergy, radio-immunomodulation, tumor immune microenvironment

## Abstract

FLASH radiotherapy (FLASH-RT), characterized by ultra-high dose rate irradiation (≥40 Gy/s), has generated substantial interest in radiation oncology due to its reported capacity to preserve normal tissues while maintaining tumor control—a phenomenon termed the “FLASH effect.” This review synthesizes current advances in FLASH-RT, emphasizing its core biological mechanisms, including radiolytic oxygen depletion, free radical recombination, immune modulation, DNA integrity preservation, and vascular normalization. Furthermore, we explore the synergistic potential of FLASH-RT combined with immunotherapy strategies such as immune checkpoint inhibitors (ICIs), CAR-T cells, and cancer vaccines. Preclinical evidence indicates that FLASH-RT reduces lymphodepletion, enhances CD8^+^ T cell infiltration, and downregulates immunosuppressive factors (e.g., TGF-β and PD-L1), thereby overcoming limitations of conventional radiotherapy (CONV-RT) and amplifying antitumor immunity. Early-phase clinical trials (e.g., FAST-01, IMPULSE) have demonstrated preliminary safety and efficacy, yet challenges remain in mechanistic elucidation, technological standardization, and regulatory approval. Current evidence is predominantly derived from preclinical models, and conflicting data exist regarding tissue-specific FLASH effects (e.g., protection absent in gonads). Moreover, the synergistic mechanisms with immunotherapy remain largely hypothetical, supported by limited *in vivo* studies. By addressing these barriers through interdisciplinary collaboration, FLASH-RT may eventually advance precision radio-immunotherapy, but substantial translational research is still required before it can supplant conventional paradigms.

## Highlights

1. Technique: Uses ultra-high dose rate (≥40 Gy/s) delivery in ultra-short time, significantly reducing normal tissue toxicity while maintaining tumor control compared to CONV-RT (“FLASH effect”).
2. Mechanisms: Involves rapid oxygen depletion (reducing oxidative damage), radical recombination, DNA protection, vascular modulation, and regulated cell death pathways.
3. Immunological Advantage: Optimizes tumor immune microenvironment (e.g., increases CD8^+^ T cell infiltration, promotes M1 macrophage polarization, suppresses harmful inflammation and PD-L1), avoiding certain immunosuppressive effects of CONV-RT (e.g., recruitment of Tregs, MDSCs).
4. Combination Potential: Synergizes with immune checkpoint inhibitors, CAR-T, cancer vaccines, or oncolytic viruses to enhance anti-tumor immunity.
5. Status & Challenges: Early trials (e.g., FAST-01, IMPULSE) demonstrate safety, but biological mechanisms and optimal parameters require further investigation.

## 1 Introduction

Tumor radiotherapy (RT) is a cornerstone of cancer treatment, serving as a primary therapeutic modality for nearly all types of tumors—either alone or in combination with surgery and chemotherapy (1). The ionizing radiation used in radiotherapy includes α, β, and γ rays emitted by radioactive isotopes, as well as X-rays, electron beams, proton beams, and other charged or uncharged particle beams produced by medical accelerators or irradiation devices (2). Although conventional radiotherapy (CONV-RT) is effective in eradicating tumors, its efficacy is often limited by collateral damage to surrounding normal tissues, resulting in radiotoxicity in organs such as the skin, gastrointestinal tract, lungs, and brain (3). The severity of these adverse effects is influenced by factors such as total radiation dose, fractionation regimen, and individual radiosensitivity (4, 5).

In recent years, FLASH-RT has been proposed as a potentially valuable approach in radiation oncology, distinguished by its delivery of ultra-high dose rate (UHDR) irradiation within microseconds to milliseconds—typically exceeding 40 Gy/s (6). In contrast to CONV-RT, which employs a conventional dose rate (CDR) of less than 0.03 Gy/s, FLASH-RT has demonstrated a remarkable capacity to preserve normal tissues while achieving tumor control efficacy comparable to that of CONV-RT (7).

The most frequently cited property of FLASH-RT is its protective effect on normal tissues. This phenomenon, now widely referred to as the “FLASH effect”, was first hinted at in 1959, when Dewey and Boag observed an increase in the survival rate of hypoxic bacteria following ultra-high dose rate irradiation (8). However, it was not until 2014 that Favaudon et al. formally established the concept of FLASH-RT in mammalian systems (9). Using a mouse model of radiation-induced pulmonary fibrosis, their study showed that FLASH-RT (at dose rates ≥40 Gy/s) significantly mitigated lung fibrosis compared with CONV-RT (delivered at ≤0.03 Gy/s), thereby providing the first preclinical evidence of normal tissue protection in mammals. A detailed comparison of the advantages and disadvantages is presented in the table below (**Table 1**).

**Table 1:** Comparison of CONV-RT and FLASH-RT.

Feature	Conventional Radiotherapy (CONV-RT)	FLASH Radiotherapy (FLASH-RT)
Dose Rate	Low dose rate (0.01–0.1 Gy/s)	Ultra-high dose rate (≥ 40 Gy/s)
Treatment Time per Fraction	Long (typically several minutes)	Extremely short (sub-second, < 0.5 seconds)
Key Physical Characteristic	Continuous or fractionated irradiation; susceptible to intra-fraction motion (e.g., breathing).	Ultra-rapid delivery; virtually eliminates intra-fraction motion error.
Advantages	Mature, widely available technology. Extensive clinical data and standardized protocols. Suitable for a wide range of tumor types and depths. Well-established dosimetry and quality assurance systems.	FLASH Effect: Significant sparing of normal tissues (e.g., lung, skin, intestine) while maintaining tumor kill. Potential for safe dose escalation to overcome radioresistance. May preserve circulating immune cells, potentially enhancing synergy with immunotherapy. Reduced treatment time improves patient comfort and workflow.
Disadvantages	Higher toxicity to normal tissues (e.g., radiation pneumonitis, fibrosis). May cause lymphopenia, potentially suppressing immune response. Dose escalation limited by normal tissue tolerance.	High technical requirements for equipment (e.g., ultra-high beam current, rapid energy switching). Clinical data is still limited, lacking large-scale validation. Dosimetry and real-time monitoring techniques are not yet mature. Underlying biological mechanisms (e.g., oxygen depletion, radical recombination) are not fully understood. Currently limited to superficial/small-volume tumors (electron beams) or in developmental stages (proton/heavy ions).

The year 2019 marked a milestone with the first reported application of FLASH-RT in a human patient—a 75-year-old individual with multidrug-resistant cutaneous lymphoma. The treatment resulted in rapid tumor regression accompanied by only mild and transient toxicities, offering initial clinical evidence supporting both the safety and efficacy of FLASH-RT (10).

Driven by advancements in medical accelerator technology, research on FLASH-RT has accelerated. An increasing number of *in vitro* and *in vivo* studies have reported normal tissue-sparing effects of FLASH irradiation in various biological systems and tissue types, although with notable exceptions and unresolved variability. Despite the promising potential demonstrated in preclinical studies, it must be recognized that FLASH radiotherapy remains largely in the proof-of-concept and preclinical exploration stage, with clinical translational maturity far below that of conventional therapies. Most evidence supporting its biological effects is derived from *in vitro* experiments and animal models, and the limited early clinical trials (e.g., FAST-01/02) have only validated its feasibility without addressing the core issues of combination with complex strategies such as immunotherapy. Furthermore, the FLASH effect still faces fundamental challenges, including unclear mechanisms underlying its absence in specific tissues (e.g., gonads) and significant discrepancies in key physical parameters (e.g., dose-rate thresholds) reported across research groups, with no unified standards. Therefore, this paper, while systematically reviewing the current progress in the field, will maintain a prudent stance and objectively present both the opportunities and challenges.

## 2 Technical and biological foundations of FLASH-RT: a critical appraisal

While FLASH-RT has emerged as a promising approach with unique biological effects, a comprehensive understanding of its potential relies on dissecting two fundamental aspects. First, the types and characteristics of radiation sources underpinning FLASH delivery determine its technical feasibility and dose distribution, laying the groundwork for its clinical application. Second, unraveling the potential core mechanisms that drive FLASH’s distinct responses—from normal tissue sparing to immunomodulation—is critical to harnessing its therapeutic advantages. This chapter will delve into these two interconnected areas, starting with the radiation sources integral to FLASH and progressing to the key mechanisms thought to mediate its remarkable effects.

### 2.1 Radiation sources for FLASH delivery and their technical limits

The selection of radiation sources is one of the core links in the implementation and clinical translation of FLASH-RT technology. FLASH-RT relies on the unique biological effects produced by ultra-high dose rates (usually defined as ≥40 Gy/s), which significantly reduce normal tissue damage while maintaining tumor control effects. The full play of this effect highly depends on whether the radiation source can stably provide the required dose rate, sufficient penetration depth, and precise dose distribution. Currently, the radiation sources used in FLASH-RT research mainly include four types: electron beams, X-rays, protons, and heavy ions. Different radiation sources have distinct characteristics in terms of particle type, energy range, dose rate improvement potential, and clinical adaptation scenarios, and their application advantages and challenges in FLASH mode also show obvious differences. Based on the core characteristics of each radiation source and combined with the technical requirements of FLASH-RT, the application adaptability of the four main radiation sources is summarized below, and the specific contents are shown in the following table (**Table 2**). It is important to emphasize that the biological consequences of FLASH irradiation—particularly the normal tissue-sparing effect—are not intrinsic properties of the radiation source itself, but emerge from the complex interaction between ultra-high dose rate delivery and the physiological characteristics of the irradiated tissue. While electron, photon, proton, and heavy-ion FLASH each have distinct physical advantages, whether they all elicit identical biological FLASH effects remains an open question. Comparative studies using the same biological endpoint across different radiation modalities are urgently needed.

**Table 2:** Summary of characteristics and applicability of four radiation sources in FLASH radiotherapy.

Radiation source	Type energy range	Dose rate increase potential	Clinical application scenarios	FLASH application advantages	FLASH application challenges
Electron Beam	4-20 MeV	High	Superficial tumors (e.g., skin cancer)	Easy to achieve high dose rates; relatively simple equipment	Limited penetration, unsuitable for deep-seated tumors
X-ray	6-25 MV	Medium to High	Tumors at various depths; widely used	Mature technology, highly adaptable	Potential for scatter dose, risk of normal tissue damage
Proton	70-250 MeV	Medium to High	Deep-seated tumors, precise targeting (e.g., ocular, brain)	Bragg Peak effect spares normal tissue	Expensive equipment; dose rate limited by accelerator technology
Heavy Ion	100-430 MeV/u	Medium to High	Radioresistant tumors (e.g., osteosarcoma)	High LET, strong biological effect, precise targeting	Extremely expensive equipment; complex dose rate control

### 2.2 The multifactorial and unresolved biology of FLASH

Currently, the biological mechanism(s) underlying the FLASH effect remain incompletely elucidated and vigorously debated. Multiple working hypotheses have been proposed—including oxygen depletion, free radical recombination, DNA integrity preservation, vascular normalization, mitochondrial modulation, and immune protection. However, each hypothesis rests on incomplete evidence, and none can yet explain all experimental observations, particularly the puzzling tissue-specific absence of the FLASH effect. In the following subsections, we critically evaluate each hypothesis, explicitly distinguishing between empirically supported findings and speculative extensions, and highlight the key contradictions and knowledge gaps that must be resolved.

Montay-Gruel et al. provided the first *in vivo* experimental validation of the protective effect of FLASH-RT. Their experiments demonstrated that FLASH irradiation substantially mitigated tissue damage and reduced the production of reactive oxygen species (ROS) (11). A defining feature of FLASH-RT is the deposition of radiation energy within an extremely short timeframe, which induces a rapid decline in physiological oxygen levels in normal tissues. Before reoxygenation can occur, the formation of peroxyl radicals is constrained, leading to a corresponding decrease in complex DNA damage clusters and double-strand breaks (DSBs)—thereby reducing radiotoxicity to normal tissues (12). Furthermore, Rothwell et al. using a reaction-diffusion model, found that the resulting hypoxic microenvironment can enhance DNA repair efficiency, further alleviating radiation-induced damage (13). Given that chronically hypoxic tumor tissues inherently exhibit limited potential for further oxygen depletion, it is hypothesized that the difference in radiosensitivity between FLASH-RT and CONV-RT within tumor regions may be minimal; direct experimental confirmation in human tumors is lacking.

In 1969, Berry experimentally demonstrated the “FLASH” effect in HeLa and CHL-F cells, while explicitly questioning the applicability of the oxygen depletion theory in mammalian systems. He argued that within the critical dose range of 5–10 Gray (Gy), complete oxygen depletion is unattainable due to the rapid diffusion of oxygen in biological tissues, which prevents substantial oxygen consumption (14). Consequently, he hypothesized that the primary determinant of radiation-induced damage might not be oxygen depletion itself, but rather the interactions among free radicals. Despite subsequent computational models attempting to reconcile Berry’s objection with the FLASH effect, direct experimental evidence for rapid, biologically significant oxygen depletion *in vivo* remains lacking. The oxygen depletion hypothesis therefore remains a plausible but unproven mechanism, and its relative contribution compared to radical recombination or other pathways is still vigorously debated.

LaBarre et al. formally proposed the free radical recombination hypothesis in 2020 (15). They developed a physicochemical model simulating water radiolysis, oxygen consumption, and free radical reactions. Model validation revealed that under ultra-high dose-rate conditions, peroxyl radicals (ROO·) rapidly reach a steady state and undergo recombination after irradiation. This process reduces the total radical exposure (as measured by area under the curve, AUC), which aligns closely with the protective effects of FLASH-RT on normal tissues. Subsequent studies suggested that the enhanced recombination at high dose rates may be attributable to the spatial overlap of free radical trajectories. It is important to note that the FLASH effect depends not only on average dose rate but also on pulse structure parameters—including instantaneous dose rate, pulse width, pulse repetition frequency, and number of pulses per fraction. Support for this mechanism was provided by Baikalov’s Monte Carlo simulation experiments, which demonstrated that when the radiation dose exceeds 90 Gy with a pulse duration shorter than 0.5 μs, interactions among radical trajectories are significantly enhanced, further underscoring the critical role of free radical recombination in the FLASH effect (16). However, most preclinical FLASH studies use widely varying pulse parameters, and no systematic comparisons exist to determine how these parameters independently influence immune modulation or normal tissue sparing. This variability may contribute to conflicting reports in the literature and underscores the need for standardized reporting of pulse structure in future studies. Meanwhile, the radical recombination hypothesis is derived almost exclusively from Monte Carlo simulations and physicochemical models; direct experimental evidence for enhanced radical-radical interactions under FLASH conditions in living tissues is still lacking. Furthermore, this model does not readily explain why certain tissues (e.g., gonads) fail to exhibit FLASH protection, as radical recombination kinetics are expected to be similar across most soft tissues. It remains unclear whether tissue-specific differences in endogenous antioxidants, metal ion content, or repair capacity modulate the net biological consequence of altered radical chemistry.

Following the clarification of the protective role of free radical recombination in normal tissues, the research community shifted its focus toward understanding the differential responses of tumor versus normal tissues under FLASH irradiation. Labarbe hypothesized that this discrepancy might originate from insufficient antioxidant defense mechanisms in tumor cells. Based on a conceptual model integrating physical chemistry and redox metabolism, Spitz and colleagues conducted further investigations which revealed that elevated levels of free iron in tumor cells promote reactive oxygen species (ROS) generation and DNA damage via the Fenton reaction. This finding underscores the crucial role of redox metabolism in mediating the differential manifestations of the FLASH effect between tumor and normal tissues (17).

Using a murine Lewis lung cancer model, Shukla et al. (18) compared the antitumor effects of FLASH and CONV-RT. The results demonstrated that FLASH-RT significantly enhanced the infiltration of CD8^+^ cytotoxic T cells within the tumor core, while reducing the proportion of immunosuppressive regulatory T cells (Tregs). Furthermore, FLASH-RT was associated with an increased proportion of M1-type macrophages and a decreased proportion of M2-type macrophages in this model, suggesting a potential shift toward a more immunostimulatory phenotype. Whether this observation is generalizable or causally linked to tumor control remains unknown. Additionally, studies have revealed that FLASH-RT can modulate the levels of inflammatory cytokines in the bloodstream. Cunningham et al. reported that, compared to CONV-RT, FLASH-RT significantly reduced blood concentrations of the chemokine CXCL1 and transforming growth factor-β1 (TGF-β1), while increasing the ratio of granulocyte-macrophage colony-stimulating factor (GM-CSF) to granulocyte colony-stimulating factor (G-CSF) (19). These observations are intriguing but remain correlative. Whether the observed immune modulation is a cause or a consequence of normal tissue sparing is unknown, and no study has yet demonstrated that depletion of specific immune subsets abolishes the FLASH effect. Moreover, the relevance of these findings to immunologically privileged sites (e.g., gonads, brain) has not been investigated, leaving the tissue-specific failure of FLASH in gonads mechanistically unexplained.

The DNA Integrity Hypothesis, proposed by Shi et al. (20), offers a novel perspective for understanding the FLASH effect. The core tenet of this hypothesis is that the protective effect of FLASH-RT stems from its superior preservation of genomic DNA integrity. Due to its prolonged irradiation time, DNA damage accumulates continuously in CONV-RT during dose delivery, thereby exacerbating genomic instability. In contrast, the ultra-rapid dose delivery of FLASH-RT minimizes additional DNA damage generated during irradiation. The authors proposed that this mechanism could explain how FLASH-RT maintains tumor-killing efficacy comparable to CONV-RT while appearing to better protect normal tissues. This explanation, however, rests on the unproven assumption that tumor cells are intrinsically insensitive to the DNA-protective effect. In a specific investigation into the protective effects of X-ray FLASH combined with anti-PD-L1 therapy, Shi et al. (20) observed that, compared to CONV-RT, FLASH-RT significantly attenuated intestinal pyroptosis and effectively preserved crypt structures. Mechanistically, the authors proposed that this protection arises because FLASH-RT generates fewer cytoplasmic double-stranded DNA fragments, which may blunt the cGAS–STING-mediated proinflammatory response. This interpretation is derived from a single intestinal model and has not been validated in other tissues or tumor types; whether it represents a general FLASH mechanism remains to be determined.

The Vascular Normalization Hypothesis was first systematically articulated by the Marie-Catherine Vozenin team in a landmark review published in *Nature Reviews Cancer* in 2019, establishing a pivotal theoretical framework for understanding the distinctive biological effects of FLASH-RT (21). In their prior experimental work, when mouse lungs were irradiated using FLASH-RT at a single dose of 15 Gy and a dose rate ≥40 Gy/s, the integrity of vascular endothelial cells was markedly preserved. In contrast, CONV-RT delivered at the same dose but a dose rate of 0.1 Gy/s induced extensive vascular damage. This finding constituted the core empirical evidence underpinning the hypothesis (9).

In terms of specific observational indicators, CONV-RT resulted in a significant increase in vascular permeability (as evidenced by plasma protein leakage), apoptosis in over 50% of endothelial cells (Caspase-3^+^), marked upregulation of adhesion molecules ICAM-1 and VEGF, and a substantial reduction in pericyte coverage (indicating loss of vascular structural support). In contrast, FLASH-RT maintained normal vascular permeability, limited endothelial cell apoptosis to less than 5%, prevented significant changes in adhesion molecule expression, and preserved normal pericyte coverage. These findings have been interpreted as evidence that FLASH-RT may protect vascular structure and function, potentially contributing to maintenance of blood-tissue barrier integrity and inhibition of inflammatory cascades (22, 23). Direct causal evidence linking vascular protection to overall normal tissue sparing is still lacking.

Yuqi Ma et al. (24) proposed that mitochondria serve as key regulators of cell death pathways and, upon radiation exposure, influence cellular fate through the release of biomolecules. On one hand, mitochondria are central to intrinsic apoptosis: radiation induces mitochondria to generate excessive reactive oxygen species (ROS), triggering mitochondrial outer membrane permeabilization (MOMP) mediated by the pro-apoptotic proteins BAX/BAK. This leads to the release of cytochrome c (cyt c) into the cytoplasm, where it binds to APAF-1 to form the apoptosome, activating the caspase cascade and resulting in non-inflammatory apoptosis. On the other hand, MOMP also facilitates the leakage of mitochondrial DNA (mtDNA) into the cytoplasm, which activates the cGAS–STING pathway, induces typeIinterferon (IFN-I) release, and initiates an inflammatory response. However, apoptosis-related caspases can suppress this inflammatory process to prevent excessive immune activation. In normal tissues, the former pathway (non-inflammatory apoptosis) predominates, whereas in tumor tissues—due to the Warburg effect (glycolysis dependence)—mitochondrial function is impaired, resulting in reduced cyt c release and thus a shift toward the latter inflammatory response.

A critical, often underappreciated observation in FLASH radiobiology is the marked tissue-specific variability of the FLASH effect. While robust normal tissue sparing has been reproducibly demonstrated in lung, skin, intestine, and brain, no protective effect was detected in mouse gonads. This negative finding is not attributable to insufficient statistical power or dosimetric error, and it has profound implications for mechanistic theory. The tissue-specific failure of the FLASH effect demonstrates that no current hypothesis is universally valid. Instead, the FLASH effect likely represents a convergence of multiple, tissue-dependent mechanisms whose relative contributions vary according to baseline physiological parameters (oxygenation, antioxidant capacity, mitochondrial density, immune status, vascular phenotype).

**Figure 1:**
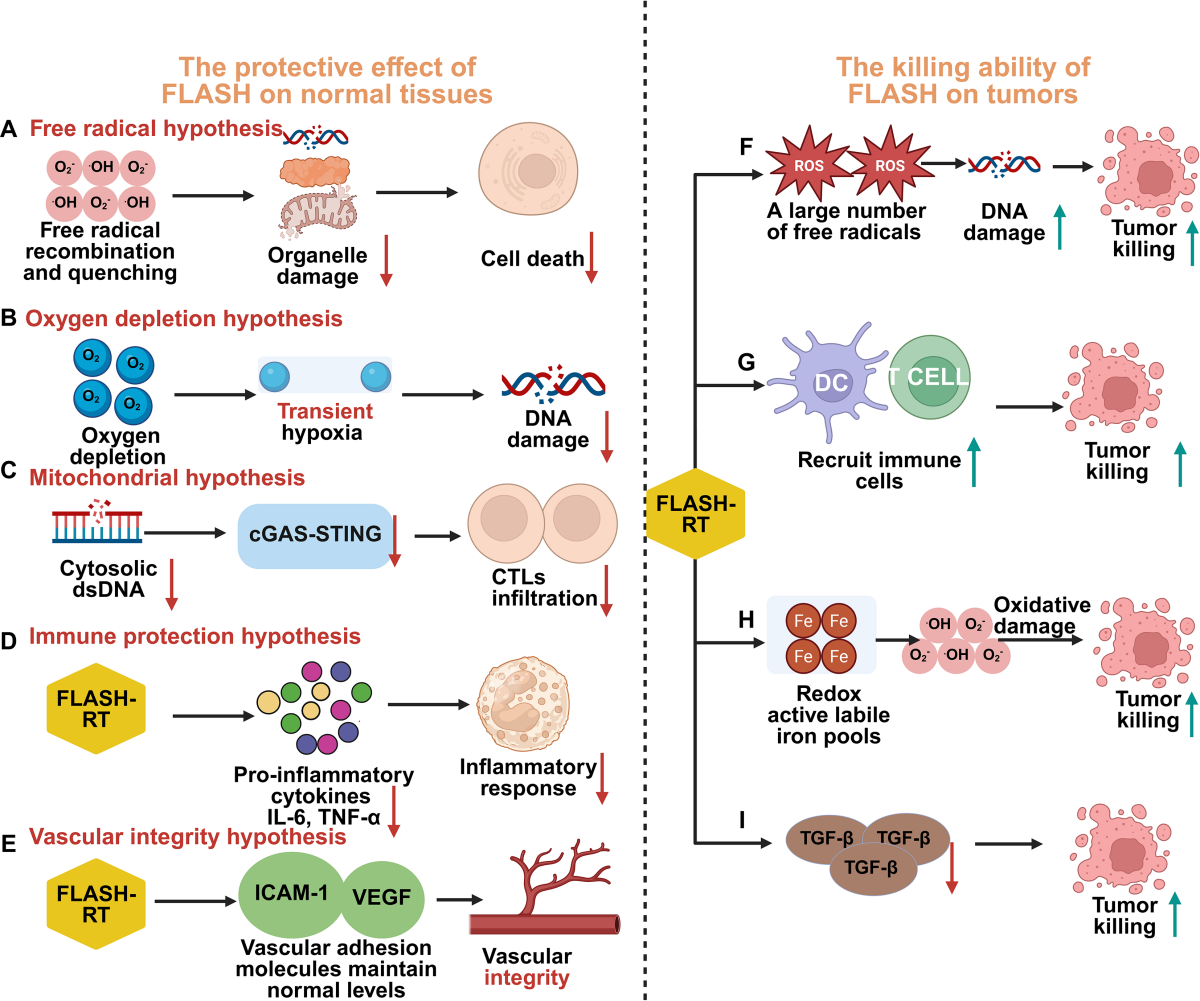
Mechanistic Insights into the FLASH Radiotherapy (FLASH-RT) Effect. Left Panel: Protective Effect on Normal Tissues: **(A)** Free Radical Hypothesis: Ultra-high dose rates promote rapid free radical recombination and quenching, reducing organelle damage and subsequent cell death in normal tissues. **(B)** Oxygen Depletion Hypothesis: FLASH-RT induces transient hypoxia through rapid oxygen consumption, mitigating oxidative DNA damage. **(C)** Mitochondrial Hypothesis: FLASH-RT may reduce the activation level of the cGAS-STING pathway by reducing cytoplasmic double-stranded DNA (dsDNA), thereby affecting the infiltration of cytotoxic T lymphocytes (CTL) and reducing the inflammatory response of normal cells. **(D)** Immune Protection Hypothesis: FLASH-RT preserves circulating immune cells and hematopoietic stem cell function, modulates the tumor immune microenvironment (TIME) by enhancing CD8^+^ T cell infiltration and M1 macrophage polarization, and reduces radiation-induced inflammation (e.g., TGF-β, CXCL1) and PD-L1 expression. **(E)** Vascular Integrity Hypothesis: FLASH-RT maintains normal levels of vascular adhesion molecules (ICAM-1, VEGF), preserving vascular integrity and reducing endothelial damage. Right Panel: Enhanced Tumor-Killing Ability: **(F)** Free Radical Accumulation: In tumors, FLASH-RT generates a large number of free radicals, causing significant DNA damage and tumor cell killing. **(G)** Immune Cell Recruitment: FLASH-RT promotes the recruitment of immune cells, including dendritic cells (DCs) and T cells, enhancing anti-tumor immune responses. **(H)** Redox Active Labile Iron Pools: FLASH-RT may exploit elevated redox-active labile iron pools in tumors, exacerbating oxidative damage. **(I)** TGF-β Inhibition: FLASH-RT downregulates TGF-β signaling, further contributing to tumor cell killing. This figure highlights the multifaceted mechanisms by which FLASH-RT achieves selective protection of normal tissues while enhancing tumor control, paving the way for synergistic combination therapies with immunotherapy.

Moving forward, mechanistic studies should deliberately compare protected versus non-protected tissues (e.g., lung vs. gonad) using multi-omics and functional assays, rather than focusing exclusively on classical model systems where the effect is robust. Identifying the critical determinants of tissue susceptibility will not only deepen mechanistic understanding but also guide clinical patient selection and dose optimization (**Figure 1**).

## 3 Comparison of the immunological effects of traditional radiotherapy and FLASH - RT in cancer treatment

Having already underscored the prominent advantages of FLASH-RT in oncology, the focus now shifts to its synergy with immunotherapy. To contextualize this synergistic strategy clearly, subsequent sections will first examine the dual effects of CONV-RT, then delve into FLASH’s potential biological and immunological merits.

### 3.1 The double-edged sword of conventional radiotherapy and immunity

CONV-RT presents a contrasting dynamic in its interaction with immunity—one widely recognized as a “double-edged sword.” This duality, which encompasses both beneficial anti-tumor immune effects and unintended pro-tumor immune consequences, not only highlights the limitations of conventional approaches but also provides critical context for appreciating FLASH’s unique value. It is this dual nature of CONV-RT’s interplay with immunity that will be unpacked in the sections ahead.

### 3.2 Activate anti-tumor immunity

In activating anti-tumor immune responses, radiotherapy primarily exerts its core function by inducing immunogenic cell death (ICD) (25, 26). When ionizing radiation penetrates tumor cells, it directly causes DNA double-strand breaks (DSBs). This damage activates DNA damage response (DDR) pathways—such as those mediated by ATM/ATR and Chk1/2—which not only signal the cell to initiate repair or apoptosis but also serve as a key trigger for ICD (27, 28).

Concurrently, reactive oxygen species (ROS) generated by radiotherapy damage intracellular proteins and trigger endoplasmic reticulum (ER) stress (29), thereby activating the unfolded protein response (UPR) (30); Upon activation of the PERK–eIF2α pathway (31), phosphorylated eIF2α inhibits the translation of most proteins while specifically upregulating the transcription factor ATF4. ATF4 further induces the expression of CHOP, ultimately promoting the translocation of calreticulin (CRT) from the ER lumen to the cell surface. As a potent “eat-me” signal, CRT specifically binds to the CD91 (LRP1) receptor on dendritic cells (DCs), markedly enhancing their phagocytic capacity toward tumor cells (31). Meanwhile, the IRE1α–XBP1 pathway upregulates the expression of chaperone proteins, providing essential support for the subsequent processing of tumor antigens (32).

During this process, tumor cells also release a suite of damage-associated molecular patterns (DAMPs), establishing a comprehensive immune activation network (33): ATP is extensively leaked into the extracellular space due to damage to the plasma and mitochondrial membranes. On one hand, it activates the NLRP3 inflammasome via P2X7 receptors on dendritic cells (DCs) and macrophages, promoting the maturation and release of IL-1β and IL-18 to amplify local inflammation (34); on the other hand, it acts as a chemotactic signal through the P2Y2 receptor to directionally recruit antigen-presenting cells (APCs) to the tumor site (35). The nuclear protein HMGB1 is passively released during the late stages of cell death and initiates signaling cascades by binding to Toll-like receptor 4 (TLR4) and the receptor for advanced glycation end products (RAGE) on DCs. Activation of the TLR4–MyD88 pathway induces nuclear factor kappa-B (NF-κB) and mitogen-activated protein kinase (MAPK) signaling, which not only promotes DC maturation—upregulating molecules such as MHC class II, CD80/86, and CD40—but also stimulates the secretion of proinflammatory cytokines including TNF-α, IL-6, and IL-12. Concurrently, it enhances the capacity of DCs to phagocytose dead cell debris and process antigens (36). Heat shock proteins (HSP70, HSP90, gp96) bind to tumor antigen peptides to prevent their degradation and are subsequently recognized and internalized by APCs via receptors including CD91, TLR2/4, SREC-I, and LOX-1, markedly enhancing cross-presentation. This process enables exogenous antigens to be presented to CD8^+^ T cells via MHC class I molecules—a pivotal step in activating cytotoxic T lymphocytes (37). Ultimately, ICD transforms dying tumor cells into “*in situ* vaccines,” robustly activating DCs and initiating adaptive immune responses (38).

In addition to directly killing tumor cells and releasing a substantial number of tumor-associated antigens (TAAs), radiotherapy also liberates neoantigens generated by unique mutations within tumor cells. These neoantigens possess stronger immunogenicity and provide more precise “targets” for immune recognition and attack (39). Meanwhile, radiotherapy orchestrates the activation and migration of dendritic cells (DCs) as well as the priming of T cells through multiple coordinated signals. Damage-associated molecular patterns (DAMPs), such as HMGB1 binding to TLR4 and heat shock proteins (HSPs) engaging TLR2/4, deliver the first signal for DC maturation (40). Inflammatory cytokines induced by radiotherapy, including TNF-α, IL-1β, and IFN-α/β, serve as the second signal (41), and together these signals synergistically promote DC maturation.

Under radiotherapy stimulation, tumor and stromal cells secrete a variety of chemokines that form a precise “recruitment network” for immune cells: CCL2 recruits monocytes via CCR2 (42), CCL5 attracts T cells through CCR5 (43), CCL20 draws immature DCs via CCR6 (44), CXCL9/10 recruit natural killer (NK) cells and T cells by binding to CXCR3, and CXCL16 recruits T cells via CXCR6. Within lymph nodes, mature DCs activate CD8^+^ T cells via MHC-I–TCR interactions and CD4^+^ T cells through MHC-II–TCR engagement, delivering the first antigen-specific signal. They provide the second costimulatory signal through CD80/CD86 binding to CD28, and the third polarizing signal via cytokine secretion (e.g., IL-12). Ultimately, this process activates tumor antigen-specific CD8^+^ cytotoxic T lymphocytes (CTLs) and CD4^+^ helper T cells, particularly those of the Th1 subtype.

When moderate-dose/fractionated radiotherapy is administered, it initiates a transient “vascular normalization window”: it inhibits pro-angiogenic VEGF signaling (45), while simultaneously upregulating vessel-stabilizing molecules such as Angiopoietin-1. This leads to reduced vascular leakage, improved intratumoral blood flow and oxygenation, and decreased interstitial fluid pressure (46). Concurrently, radiotherapy induces activated T cells and NK cells to produce IFN-γ (47), which acts synergistically with TNF-α—directly induced by radiation—to significantly upregulate adhesion molecules such as ICAM-1 and VCAM-1 on endothelial cells (47). These changes enable activated effector T cells to firmly adhere to endothelial ICAM-1/VCAM-1 via their surface integrins LFA-1/VLA-4, facilitating successful extravasation into the tumor parenchyma and thereby overcoming the problem of immune exclusion (“inability to enter”) (48–51).

DNA damage and activation of the IFN-γ/JAK–STAT pathway significantly upregulate the expression of MHC class I molecules on the surface of tumor cells, markedly enhancing their capacity to present endogenous antigenic peptides to CD8^+^ T cells. These mechanisms can even partially reverse MHC-I downregulation resulting from genetic defects—a common immune evasion strategy employed by tumors. Furthermore, ER stress/UPR and IFN signaling upregulate key components of the antigen-processing machinery, including antigen peptide transporters (TAP1/TAP2) and immunoproteasome subunits (LMP2/LMP7), thereby accelerating the production and translocation of antigenic peptides. Concurrently, radiotherapy induces tumor cells to upregulate death receptors such as Fas (CD95) and TRAIL receptors (DR4/DR5), rendering them more susceptible to killing mediated by FasL and TRAIL expressed by cytotoxic T lymphocytes (CTLs).

In terms of innate immune activation, damage-associated molecular patterns (DAMPs) released by radiotherapy—such as HMGB1, HSPs, DNA, and RNA—activate innate immune cells including macrophages, dendritic cells (DCs), and natural killer (NK) cells through pattern recognition receptors (PRRs). Among these, the cGAS–STING pathway plays a particularly critical role: radiotherapy-induced leakage of mitochondrial or nuclear DNA leads to the accumulation of cytoplasmic DNA, which activates the DNA sensor cGAS (52–54) and catalyzes the synthesis of the second messenger cyclic dinucleotide cGAMP (55, 56). Upon binding of cGAMP to the endoplasmic reticulum protein STING, TBK1 is recruited and phosphorylates IRF3 (57, 58), thereby initiating the transcription of typeIinterferons (IFN-α/β) and CXCL10. TypeIinterferons, in turn, broadly activate DCs, NK cells, and T cells, and upregulate the expression of MHC molecules (59–62).

In the context of radiotherapy, both canonical and non-canonical NF-κB signaling pathways play essential roles in cGAS–STING-mediated typeIinterferon expression, alongside IRF3. Generally, NF-κB is activated through canonical and non-canonical pathways. The canonical pathway mediates the activation of NF-κB1 p50, RELA, and c-REL, while the non-canonical pathway selectively activates NF-κB members sequestered by p100, primarily NF-κB2 p52 and RELB. Beyond reactive oxygen species generation, ionizing radiation-induced DNA damage signaling also activates the NF-κB pathway, predominantly through the IKK-dependent canonical signaling route (63). IFN-β expression is largely dependent on enhanceosome assembly, where canonical NF-κB acts synergistically with IRF3 and other components to maximize IFN-β gene transcription (64, 65). Abe et al. observed that partial silencing of canonical NF-κB expression via RNA interference (RNAi) reduced IFN-β production by 50% in primary mouse embryonic fibroblasts (57). Similarly, both canonical NF-κB and IRF3 are required for typeIinterferon induction in dendritic cells stimulated by irradiated tumor cells following ionizing radiation (IR) (66). This aligns with another study demonstrating that impaired canonical NF-κB signaling may attenuate radiation-induced antitumor immunity (66). In contrast, it has been reported that the non-canonical pathway may suppress ionizing radiation-induced interferon release in dendritic cells with activated STING signaling. Research suggests that cancer cells exposed to ionizing radiation may promote activation of the non-canonical NF-κB pathway in dendritic cells through the aforementioned mechanisms. Taken together, in the setting of radiotherapy, canonical and non-canonical NF-κB signaling pathways may play opposing roles in regulating cGAS–STING-induced typeIinterferon expression.

Meanwhile, radiotherapy activates key transcription factors—including NF-κB, AP-1, and STAT—stimulating tumor cells, immune cells, and stromal cells to secrete a spectrum of proinflammatory cytokines such as TNF-α, IL-1β, IL-6, IL-12, IFN-γ, CXCL9, and CXCL10. This collective response forms a robust “immune activation network” that not only recruits additional immune cells to the tumor site but also promotes T cell proliferation and differentiation, directly inhibits tumor growth, and enhances the functionality of antigen-presenting cells (APCs).

### 3.3 Activate pro-tumor immunity

However, the efficacy of radiotherapy (RT) does not always align with expectations; on the contrary, the immunomodulatory effects induced by RT represent a double-edged sword—enhancing systemic antitumor immune responses while simultaneously promoting immunosuppression to a certain extent (67, 68).

Regulatory T cells (Tregs) are a subset of lymphocytes characterized by the expression of the transcription factor FOXP3, and their presence is critical for the maintenance of immune homeostasis and self-tolerance (69). The recruitment and activation of Tregs represent a classic example of such immunosuppressive effects. Furthermore, Oweida and colleagues elucidated the mechanism through which radiation regulates Treg cells using an orthotopic mouse model of head and neck cancer (HNC) (70). They demonstrated that signal transducer and activator of transcription 3 (STAT3)—a key upstream regulator of FOXP3 (71) —promotes the radiation-induced conversion of conventional T cells into Treg cells. Moreover, combining STAT3 inhibition with radiotherapy significantly remodels the tumor microenvironment: it reduces the abundance of Tregs, myeloid-derived suppressor cells (MDSCs), and M2-type macrophages, while increasing the infiltration of effector T cells and M1-type macrophages, thereby enhancing the efficacy of radiotherapy (72).

Similarly, myeloid-derived suppressor cells (MDSCs) are markedly activated by radiotherapy: factors induced by radiation therapy—such as GM-CSF, G-CSF, IL-6, VEGF, and PGE_2_—promote the expansion of MDSCs in the bone marrow and facilitate their recruitment into tumor tissues. In addition, soluble mediators including TGF-β, IL-10, and IL-4/13 (which also contribute to M2 macrophage polarization) further enhance the immunosuppressive activity of MDSCs (73–75).

Following radiotherapy, tumor-associated macrophages (TAMs) exhibit a tendency to polarize toward the M2 phenotype. Radiation-induced factors such as IL-4, IL-10, IL-13, TGF-β, M-CSF, and PGE_2_, in conjunction with the hypoxic tumor microenvironment, strongly promote the expression of M2 markers—including CD206, CD163, and ARG1—in macrophages (76–78). These M2-type TAMs secrete immunosuppressive mediators such as TGF-β, IL-10, and PGE_2_, which inhibit T cell function and impair dendritic cell (DC) maturation (79).

TGF-β plays a pervasive role in the immunosuppressive processes following radiotherapy (80). Multiple cellular components within the irradiated tumor microenvironment—including activated tumor cells, cancer-associated fibroblasts (CAFs), Tregs, M2-type TAMs, and MDSCs—secrete TGF-β (81). However, TGF-β is initially present in a latent complex form (LTGF-β) and requires activation through mechanisms involving integrins (such as αvβ6 and αvβ8), proteases (e.g., MMP2/9, TSP-1), or reactive oxygen species (ROS) and acidic conditions to become biologically active (82).

Activated TGF-β exerts its immunosuppressive effects through multiple mechanisms: it impairs the function of cytotoxic T lymphocytes (CTLs) and natural killer (NK) cells by downregulating the expression of perforin, granzyme, and IFN-γ, and reduces the level of the activating receptor NKG2D (83); it promotes the differentiation of naïve CD4^+^ T cells into inducible Tregs (iTregs) and sustains the stability and function of natural Tregs (nTregs) (84); it suppresses the maturation, migration, and antigen-presenting capacity of dendritic cells (DCs), while inducing DCs to produce IL-10 and indoleamine 2,3-dioxygenase (IDO) (85); it activates and recruits myeloid-derived suppressor cells (MDSCs), enhancing their immunosuppressive activity (86); it drives macrophage polarization toward the M2 phenotype; it strongly activates cancer-associated fibroblasts (CAFs), promoting the deposition of extracellular matrix (ECM) components such as collagen to form a physical barrier that impedes T cell infiltration—concurrently, it increases tissue stiffness to disrupt immune cell function and serves as a storage pool for sustained TGF-β release; it induces epithelial-mesenchymal transition (EMT) in tumor cells, enhancing their invasive and metastatic potential while reducing immunogenicity through mechanisms such as MHC-I downregulation; and it antagonizes the upregulation of ICAM-1 and VCAM-1 by IFN-γ/TNF-α, thereby hindering T cell extravasation.

Hypoxia represents one of the core features of the tumor microenvironment following radiotherapy. In the early phase, oxygenation may transiently improve due to vascular normalization; however, this is ultimately superseded by more severe hypoxia resulting from vascular damage or rapid tumor proliferation. Under these conditions, stabilized HIF-1α expression upregulates PD-L1 on tumor cells, promotes CD73 expression to enhance adenosine production, increases glycolytic flux leading to lactic acid accumulation, drives M2 macrophage polarization and MDSC functionality, and directly inhibits T cell function—particularly that of cytotoxic T lymphocytes (CTLs), which are highly dependent on aerobic glycolysis (87–89).

Concurrently, hypoxia and enhanced glycolysis jointly contribute to the accumulation of lactic acid and protons (H^+^), resulting in an acidotic microenvironment that suppresses the proliferation, cytotoxicity, and cytokine production of T and NK cells (90).

Nutrient deprivation also plays a significant role: the high glycolytic activity of tumor cells and immunosuppressive cells (Tregs, MDSCs) consumes large quantities of glucose, leading to impaired effector T cell function due to energy deficiency—as these cells also rely heavily on glycolysis (91). IDO1/TDO (primarily expressed by DCs, macrophages, and tumor cells) metabolizes tryptophan into kynurenine, which activates the aryl hydrocarbon receptor (AhR), induces Treg differentiation, inhibits CTL function, and promotes T cell apoptosis (92). Meanwhile, Arg1 (expressed by MDSCs, M2-TAMs, and tumor cells) extensively depletes arginine, further suppressing T cell activity (93).

**Figure 2:**
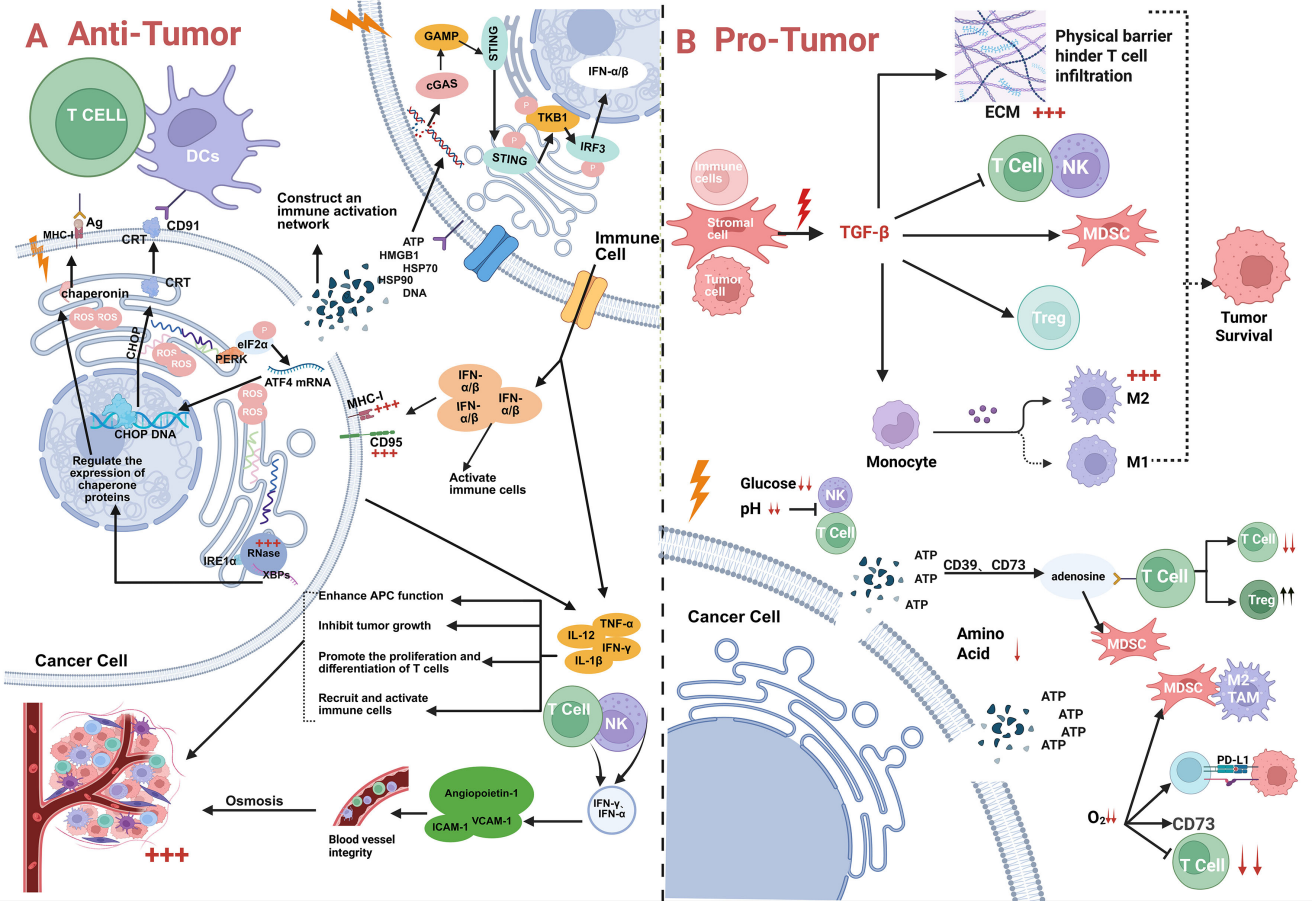
The double-edged sword of conventional radiotherapy and immunity. **(A)** Radiation therapy exerts dual effects on tumors: both antitumor and pro-tumor.When exhibiting antitumor effects, radiation therapy induces tumor cell DNA release to activate the cGAS-STING pathway, thereby stimulating IFN-α/β production. This not only activates immune cells but also induces the expression of antigen-related molecules on tumor cell surfaces, such as MHC-I and CD95. Simultaneously, it triggers endoplasmic reticulum stress within tumor cells, regulating CHOP and chaperone protein expression and translocation. This increases the presentation of molecules like MHC-I and CRT on the tumor cell surface, enabling effective recognition by immune cells. Under radiotherapy, cells secrete cytokines like TNF-α, IL-2, and IFN-γ to inhibit tumor growth, promote T-cell proliferation and differentiation, and recruit activated T cells and NK cells. Additionally, radiotherapy influences vascular integrity-related molecules such as Angiopoietin-1, ICAM-1, and VCAM-1, maintaining vascular stability and facilitating effective immune cell infiltration. Together, these mechanisms activate the tumor immune microenvironment, exerting an antitumor effect. **(B)** On the other hand, tumor promotion involves multiple mechanisms. First, TGF-β secreted by tumor or stromal cells under radiation therapy stimulation induces massive extracellular matrix (ECM) production, forming a physical barrier that impedes T cell infiltration. Simultaneously, it enhances the activity of immunosuppressive cells like MDSCs and Tregs while suppressing T cell and NK cell function to favor tumor survival. Second, increased glucose levels and altered pH in the tumor microenvironment impair NK and T cell function while disrupting monocyte differentiation. Furthermore, the metabolism of ATP to adenosine via CD39 and CD73 inhibits T cells and enhances MDSC-mediated immunosuppression. Third, hypoxia in the tumor microenvironment induces CD73 expression. The resulting adenosine suppresses T cell function while enhancing the immunosuppressive effects of MDSCs and M2-TAMs. Concurrently, it increases the expression of immune checkpoints like PD-L1, enabling tumor cells to evade immune cell killing and further driving tumor progression.

Adenosine accumulation represents another major immunosuppressive mechanism: radiotherapy induces substantial ATP release into the extracellular space, where it is hydrolyzed to AMP by CD39 (ENTPD1) and subsequently to adenosine by CD73 (NT5E) (94). Adenosine binding to A_2_A receptors—highly expressed on T and NK cells—activates the cAMP-PKA pathway, strongly inhibiting TCR signaling, cytokine production, cytotoxicity, and proliferation; this promotes T cell anergy and apoptosis while enhancing Treg functionality. Additionally, via A_2_B receptors on myeloid cells, adenosine facilitates MDSC expansion and IL-10 production (95) (**Figure 2**).

### 3.4 Preclinical observations on immune effects of FLASH-RT

Both CONV-RT and FLASH-RT are capable of initiating antitumor immune responses through shared fundamental mechanisms, including immunogenic cell death (ICD), release of damage-associated molecular patterns, activation of the cGAS-STING pathway, and subsequent priming of tumor-specific T cells. However, the ultra-high dose rate delivery of FLASH may differentially modulate specific immunological parameters—particularly those related to lymphocyte preservation, vascular integrity, and compensatory immune checkpoint upregulation—thereby potentially offering a distinct, though not inherently “superior”, immunological profile.

### 3.5 Preserve circulating immune cells

Peripheral blood lymphocytes exhibit high radiosensitivity, and lymphopenia represents one of the most common adverse effects of radiotherapy. Previous studies have confirmed that lymphopenia is associated with poor prognosis in cancer patients and reduced efficacy of immune checkpoint inhibitors (ICIs) (96, 97). Jin et al. (6) conducted an exploratory computational study employing a simplified linear-quadratic model to estimate the extent of killing of circulating immune cells under ultra-high dose rate (FLASH) versus conventional dose rate irradiation. The authors proposed that by delivering a high radiation dose within an extremely short time window, FLASH-RT primarily targets immune cells within the irradiated volume, thereby sparing a significant proportion of circulating lymphocytes (6). Although this work provides a valuable conceptual framework for understanding the FLASH effect, it does not offer direct or conclusive evidence regarding specific biological mechanisms—particularly given that the computational model relies on a series of assumptions and parameter estimates that may not fully capture the complexity of *in vivo* immune responses to radiation. The actual radiosensitivity of immune cells can vary considerably among individuals and across species, and the α/β values used in the model entail inherent uncertainties. Further empirical validation is required to substantiate these theoretical mechanisms.

Furthermore, Jansen et al. (98) observed that the protective effect of FLASH-RT on circulating immune cells exhibited a dose-dependent enhancement, which became significant at single doses of 5 Gy, 10 Gy, 20 Gy, and 30 Gy, but was absent at FLASH radiation doses below 2 Gy. It is also widely established that circulating immune cells play a pivotal role in the therapeutic efficacy of immune checkpoint inhibitors (ICIs). Immune checkpoint molecules, including programmed death receptor 1 (PD-1), programmed death-ligand 1 (PD-L1), and cytotoxic T-lymphocyte-associated antigen 4 (CTLA-4), are expressed on the surfaces of T lymphocytes and tumor cells. Tumor cells can exploit the upregulation of these molecules to inhibit immune recognition and cytotoxic function of T lymphocytes, thereby facilitating immune evasion (99). Consequently, PD-1/PD-L1 and CTLA-4 inhibitors function by attenuating the activity of these negative immune regulatory molecules on T lymphocytes, enhancing their immune recognition capacity, and ultimately eliciting anti-tumor responses (100). The preservation of peripheral immune cell counts during FLASH-RT may thereby augment the functionality of effector lymphocytes activated by ICIs. Thus, following the delivery of a sufficient FLASH radiation dose, the preservation of circulating immune cells—particularly mature effector populations—is anticipated to contribute to a synergistic anti-tumor effect when combined with immune checkpoint inhibition.

Furthermore, Chabi et al. (101) established a robust humanized mouse model by transplanting human CD34^+^ umbilical cord blood cells into radiation-induced immunodeficient mice, demonstrating that FLASH irradiation minimizes radiation-induced damage to hematopoietic stem cells. In this study, although both FLASH and CONV-RT reduced the number of phenotypic human hematopoietic stem and progenitor cells (HSPCs), ultra-high dose rate FLASH irradiation preserved partial functionality of the irradiated HSPCs. This functional preservation supports subsequent hematopoietic regeneration and helps avoid both short-term depletion and long-term suppression of circulating immune cells (102). Therefore, the potential mechanism underlying FLASH-mediated protection of peripheral immune cells may involve the preservation of bone marrow hematopoietic function and HSPC activity, thereby contributing to an enhanced therapeutic response to immune checkpoint inhibitors (ICIs). However, this conclusion is drawn from a single humanized mouse study, and the translation of HSPC preservation to human ICIs efficacy remains speculative. Moreover, the dose-dependency observed in mice (protection only >2 Gy) may not directly extrapolate to human fractionation schedules, and the impact of FLASH on lymphocyte subsets (e.g., memory vs. naïve T cells) has not been characterized. Moreover, whether this effect is reproducible across different tumor models, fractionation schedules, and anatomical sites remains unknown. Carefully controlled head-to-head comparisons of FLASH vs. CONV-RT, using identical ICIs regimens and immunological endpoints, are urgently needed.

### 3.6 Activate the tumor immune microenvironment

The Tumor Immune Microenvironment (TIME) plays a pivotal role in mediating the immunomodulatory effects of radiotherapy (RT). Multiple studies have confirmed that ionizing radiation can positively stimulate anti-tumor immune responses by activating the TIME, thereby generating promising synergistic effects when combined with immune checkpoint inhibitors (ICIs) (103, 104). Recently, Kim et al. (105) compared the regulatory effects of FLASH-RT (FLASH RT) and CONV-RT on the TIME using a subcutaneous Lewis lung cancer mouse model irradiated with 15 Gy. They performed immunohistochemical staining on tumor tissue samples from mice treated with conventional or FLASH RT to detect CD31, phosphorylated myosin light chain (p-MLC), γH2AX (a marker of DNA double-strand breaks), intracellular reactive oxygen species, and immune cells including CD8α^+^ T cells (105). At 6 hours after conventional dose rate irradiation, tumor tissues exhibited vascular contraction, upregulation of p-MLC expression, and an increased number of γH2AX-positive cells. In contrast, these effects were not observed in FLASH-irradiated samples. Additionally, the authors found that MLC kinase inhibitors could suppress radiation-induced γH2AX changes *in vitro* (105). Notably, when CONV-RT was combined with MLC kinase inhibitors, effects resembling those of FLASH RT were observed—including reduced microvascular occlusion, decreased γH2AX-positive cells, and enhanced immune cell infiltration (105). The authors suggested that FLASH-RT may promote activation of the tumor immune microenvironment through modulation of the MLC signaling pathway, based on observations in a single murine tumor model (105).

The potential mechanism involving the MLC signaling pathway can be summarized as follows: The core function of this pathway is to regulate endothelial cell contractility and modulate vascular permeability. CONV-RT and FLASH-RT exhibit marked differences in their impact on this pathway. CONV-RT induces a continuous increase in reactive oxygen species (ROS), leading to elevated cytoplasmic calcium ion levels and enhanced activity of myosin light chain kinase (MLCK), which promotes phosphorylation of myosin light chain (MLC). It also activates the RhoA/ROCK pathway and inhibits myosin light chain phosphatase (MLCP), ultimately resulting in sustained MLC phosphorylation. This forces sustained endothelial cell contraction, triggering vascular leakage and tissue edema. In contrast, FLASH-RT, by virtue of its ultra-high dose rate (≥40 Gy/s), generates only a transient and controlled “ROS pulse.” It induces a mild influx of calcium ions (Ca²^+^), activating calmodulin without eliciting a pathological response. Although FLASH transiently activates ROCK kinase, it does not persistently suppress MLCP, thereby maintaining physiological MLC phosphorylation levels and preserving the structural integrity of endothelial junctions. This fundamental difference in MLC regulation underlies the distinct effects of FLASH on vascular permeability and contributes to its unique biological profile (105). While this study provides a plausible mechanistic candidate for differential TIME modulation, several caveats must be emphasized. First, the findings are derived from a single tumor model (Lewis lung carcinoma) and have not been replicated in other cancer types. Second, the conclusion that FLASH “promotes TIME activation” relative to CONV-RT is inferred from reduced vascular contraction and enhanced CD8^+^ infiltration at a single 15 Gy dose; whether this translates to improved therapeutic outcomes when combined with immunotherapy has not been tested. Finally, the MLC kinase inhibitor experiment, while elegant, represents a pharmacological mimicry of FLASH, not direct validation that MLC signaling is the causative mechanism of the FLASH effect *in vivo*.

**Figure 3:**
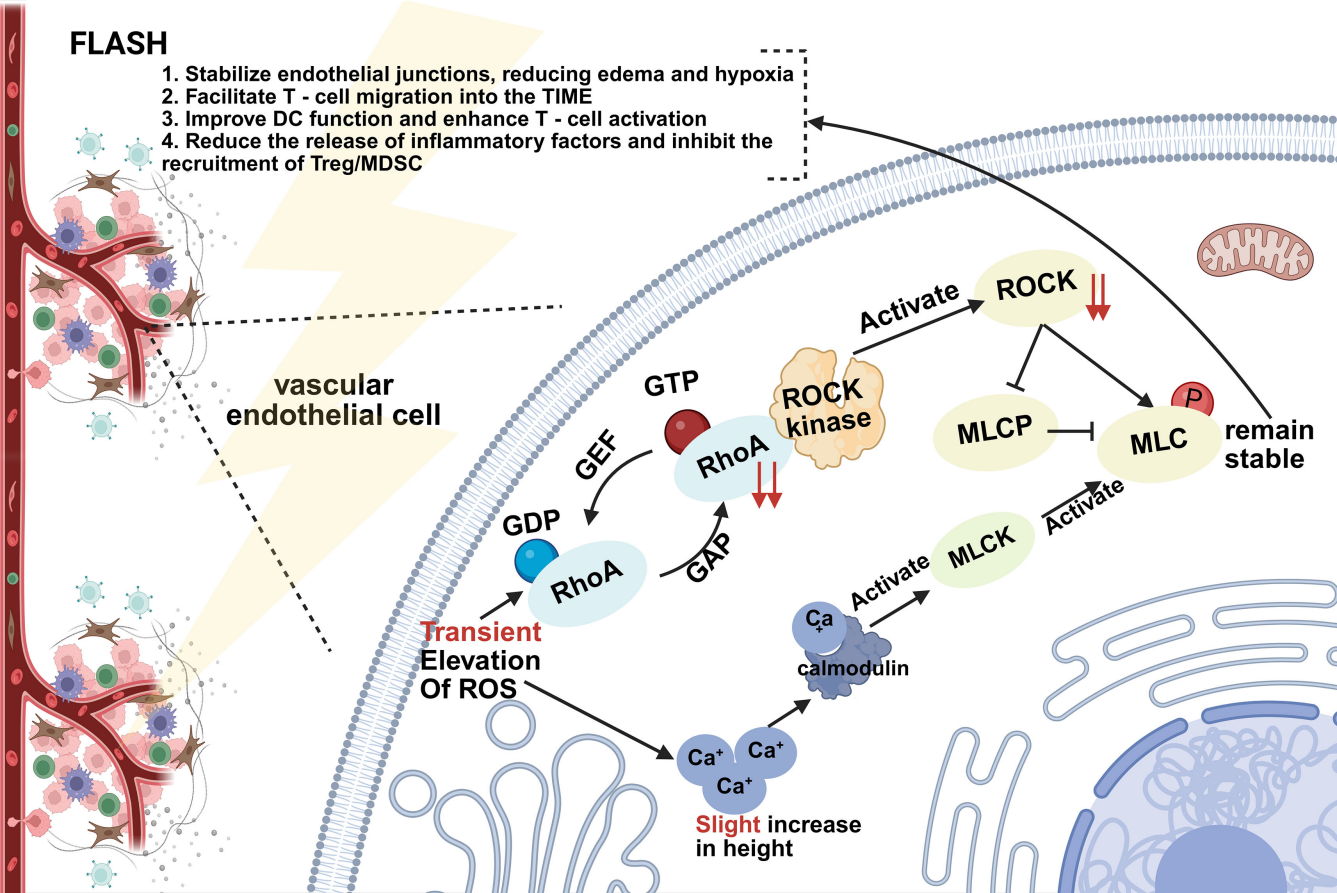
FLASH maintains vascular integrity. FLASH radiotherapy, with its ultra-high dose rate, induces only transient, controllable “transient ROS pulses.” It simultaneously activates MLCK through mild calcium ion (Ca^2+^) influx and calmodulin binding, without triggering pathological-level responses. At the ROCK/MLCP equilibrium level, FLASH radiotherapy transiently activates ROCK kinase without persistently inhibiting MLCP, maintaining stable MLC phosphorylation levels and ultimately preserving the structural integrity of endothelial cell junctions. This endothelial junction stability not only reduces edema and hypoxia but also promotes T cell migration into the tumor immune microenvironment (TIME), enhances dendritic cell (DC) function and T cell activation, while simultaneously decreasing inflammatory cytokine release and suppressing the recruitment of regulatory T cells (Treg) and myeloid-derived suppressor cells (MDSC). Thus, it exerts multifaceted effects through multiple mechanisms.

Other studies have further demonstrated that following ultra-high dose rate FLASH-RT, robust recruitment of tumor-associated macrophages (TAMs), effector T lymphocytes, and other immune cells may occur within tumor tissues. This suggests effective activation of the tumor immune microenvironment (TIME), potentially converting immunologically “cold” tumors into “hot” tumors (106–108). Such immunomodulatory effects may enhance immune recognition and antitumor efficacy when FLASH-RT is combined with immune checkpoint inhibitors (ICIs) (**Figure 3**).

### 3.7 Alleviate radiation adverse effects

Despite the significant therapeutic benefits of immune checkpoint inhibitors (ICIs), the risk of adverse events (AEs) remains a concern—particularly when combined with radiotherapy (RT). A pan-cancer meta-analysis reported that the incidence of all-grade AEs was 89.4%, and grade ≥3 AEs reached 12.4% following combined RT and immunotherapy (109). In real-world settings, the incidence of all-grade pneumonitis was 35% in patients with locally advanced non-small cell lung cancer (NSCLC) who received definitive radiotherapy followed by consolidative ICIs therapy (110).

Given the reproducibly observed protective effects of FLASH on normal tissues in preclinical models, it is plausible that combining FLASH-RT with ICIs could reduce radiotherapy-related adverse events, potentially enabling dose escalation. This remains speculative pending clinical validation. Transforming growth factor-β (TGF-β) is a key inflammatory regulator implicated in the development and progression of radiation pneumonitis and pulmonary fibrosis (111). *In vitro* experiments demonstrated that, compared to irradiation at 0.2 Gy/s, ultra-high dose rate irradiation at 1000 Gy/s significantly reduced TGF-β production in normal lung fibroblasts (IMR90) (112). In animal studies, ultra-high dose rate FLASH irradiation at 60 Gy/s markedly suppressed radiation-induced activation of the TGF-β/SMAD pathway and downregulated its downstream effector proteins compared to conventional dose rate irradiation, correspondingly reducing the incidence of pulmonary fibrosis in mice (9). Fouillard et al. (113) observed that FLASH irradiation decreased radiotherapy-induced TGF-β1 expression in pulmonary interstitial macrophages. Moreover, several studies indicate that FLASH-RT not only reduces TGF-β levels within the irradiated field but also systemically, which may help alleviate both local and systemic inflammatory injury when combined with ICIs (19).

FLASH-RT is characterized by its ultra-high dose rate (typically ≥40 Gy/s), which underlies its unique physical and biological effects, including the suppression of TGF-β production. It induces a transient and controllable reactive oxygen species (ROS) pulse, avoiding the persistent ROS accumulation seen in CONV-RT that leads to DNA damage accumulation and amplified inflammatory cascades. Thereby, FLASH downregulates TGF-β gene expression, reducing pro-fibrotic and TGF-β-synthetic signals at the source.

**Figure 4:**
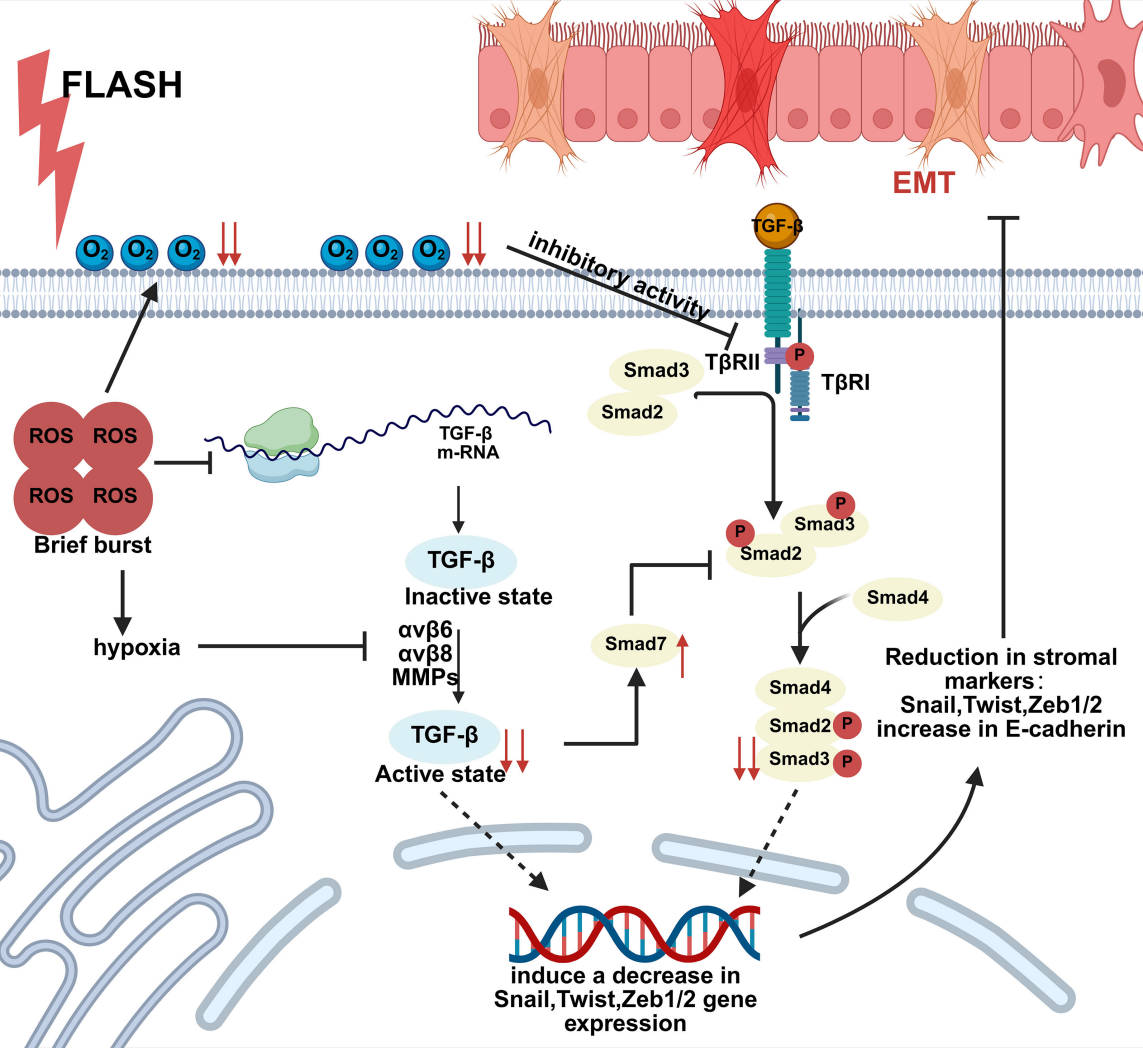
FLASH inhibits EMT. FLASH radiotherapy induces transient, controllable pulses of reactive oxygen species (ROS), avoiding the cumulative DNA damage and amplified inflammatory cascade caused by sustained ROS elevation in conventional radiotherapy, thereby reducing TGF-β gene expression. This diminishes pro-fibrotic signals and TGF-β synthesis at their source; TGF-β activation requires metalloproteinase and integrin participation. The hypoxia induced by FLASH suppresses metalloproteinase and integrin activity, blocking TGF-β activation. The hypoxic environment also reduces TGF-β receptor expression, thereby weakening downstream Smad signaling: activated TβRI phosphorylates Smad2 and Smad3, Phosphorylated Smad2/3 forms a complex with Smad4 to regulate downstream genes. Furthermore, the increased expression of Smad7 due to decreased TGF-β expression further inhibits the phosphorylation of Smad2/3. Ultimately, this induces reduced gene expression of matrix markers such as Snail, Twist, and Zeb1/2, while increasing E-cadherin expression, thereby inhibiting the epithelial-mesenchymal transition (EMT) process.

Moreover, TGF-β activation requires metalloproteinases and integrins; the hypoxic microenvironment induced by FLASH irradiation inhibits the activity of these enzymes and receptors, preventing TGF-β activation. This hypoxic environment also downregulates TGF-β receptor expression, attenuating downstream Smad signaling. Consequently, the expression of epithelial-mesenchymal transition (EMT) core transcription factors is reduced, E-cadherin levels are increased, and the EMT process is impeded. The suppression of EMT not only lowers the risk of tumor metastasis but also alleviates fibrosis in normal tissues (**Figure 4**).

Radiation-induced inflammatory damage is a complex process involving the activation of multiple immune mediators and the recruitment of various immune cells. Beyond suppressing TGF-β expression, FLASH-RT also reduces the release of numerous other pro-inflammatory cytokines, thereby potentially decreasing the risk of radiation-induced adverse events (19).

Compared to CONV-RT, FLASH-irradiated mice exhibited significantly lower levels of C-X-C motif chemokine ligand 1 (CXCL1) and granulocyte colony-stimulating factor (G-CSF) in peripheral blood, while granulocyte-macrophage colony-stimulating factor (GM-CSF) was relatively increased. Additionally, a marked reduction in early skin toxicity and late soft tissue contracture was observed in the FLASH-treated group (19).

CXCL1 plays a critical role in inflammatory immune regulation. For example, in patients with cystic fibrosis, the blood GM-CSF/G-CSF ratio has been shown to be negatively correlated with soft tissue toxicity (19). Therefore, the increased GM-CSF/G-CSF ratio following FLASH-RT further underscores its role in protecting normal tissues from immune-inflammatory injury, highlighting the enhanced safety profile of FLASH when combined with immunotherapy.

Interestingly, the immunological benefits of FLASH-RT described above—particularly its capacity to reduce cytoplasmic DNA fragments and suppress the cGAS-STING pathway in normal tissues (e.g., intestine) \[20\], while still promoting an anti-tumor immune microenvironment—present a seemingly paradoxical scenario. If the cGAS-STING pathway is a master regulator of radiotherapy-induced antitumor immunity, how can its suppression be compatible with, or even conducive to, effective immune activation against cancer? This apparent contradiction likely reflects a fundamental, dose-dependent, and tissue-specific aspect of STING biology, which is only beginning to be understood in the context of UHDR irradiation. Resolving this paradox is crucial for a coherent mechanistic understanding of FLASH radio-immunotherapy.

### 3.8 Reduce the expression of PD-L1

It has been hypothesized that FLASH-RT may downregulate PD-L1 expression through several potential, non-mutually exclusive mechanisms, although direct experimental validation in FLASH-irradiated tumors is still limited.

It has been proposed that FLASH-RT may downregulate PD-L1 expression through modulation of the IFNγ–STAT1 pathway. Direct experimental evidence for this specific mechanism under FLASH conditions is currently limited. The specific mechanism is as follows: FLASH induces transient oxygen depletion, leading to reduced mitochondrial reactive oxygen species (ROS) levels (9); it also alters the pattern of DNA damage and decreases the generation of cytoplasmic DNA fragments (20). The reduction in cytoplasmic DNA directly suppresses activation of the cGAS–STING pathway, resulting in diminished secretion of typeIinterferons (IFN-I) (41). Reduced IFN-I secretion impairs dendritic cell (DC) maturation, attenuates antigen presentation efficiency, hinders CD8^+^ T cell activation, and lowers IFNγ production (27). IFNγ activates STAT1 through a cascade of signaling events; STAT1 dimers translocate into the nucleus and bind to the PD-L1 promoter to drive its transcription (87). Consequently, decreased IFNγ expression leads to corresponding downregulation of PD-L1 (18). (This is experimentally validated: the concentration of IFNγ in tumor tissues after FLASH irradiation was 60% lower than that after CONV-RT).

Based on these observations, it has been proposed that FLASH may interfere with upstream drivers of PD-L1 expression through upstream molecular events, avoids the “compensatory PD-L1 upregulation” often triggered by CONV-RT, and achieves more direct and efficient suppression.

A second proposed mechanism involves indirect modulation of PD-L1 via the TGF-β axis (114), involving two key pathways:

Pathway 1: FLASH reduces TGF-β secretion, alleviates its sustained inhibition of CD8^+^ T cells, and decreases the expression of T cell exhaustion markers (such as TIM-3) (115, 116). Following the restoration of T cell function, the “immune pressure” on the PD-1/PD-L1 pathway is reduced, thereby indirectly inhibiting compensatory PD-L1 overexpression in tumor cells (117). Pathway 2:FLASH impedes the activation process of TGF-β by inhibiting integrin αvβ6 (118), which reduces the nuclear translocation of the transcription factor Smad3 (81). Consequently, the binding of Smad3 to the PD-L1 promoter is weakened, directly suppressing PD-L1 expression at the transcriptional level (83, 119).

This dual mechanism—combining *tumor microenvironment remodeling* and *promoter blockade*—effectively counteracts TGF-β-driven PD-L1 overexpression, simultaneously restoring T cell function and synergistically enhancing anti-tumor immune responses.

It has also been suggested, based on extrapolation from CONV-RT studies, that FLASH might induce epigenetic modifications that could lead to sustained suppression of PD-L1 expression, which are specifically manifested as follows: FLASH induces enrichment of H3K27me3—a repressive histone mark—within the PD-L1 promoter region (120), while simultaneously promoting increased DNA methylation (121, 122), while simultaneously promoting increased DNA methylation (123, 124), leading to persistent suppression of PD-1 expression. PD-1 serves as the receptor for PD-L1; reduced PD-1 expression decreases ligand–receptor engagement during PD-1/PD-L1 binding, indirectly attenuating the functional demand for PD-L1.

**Figure 5:**
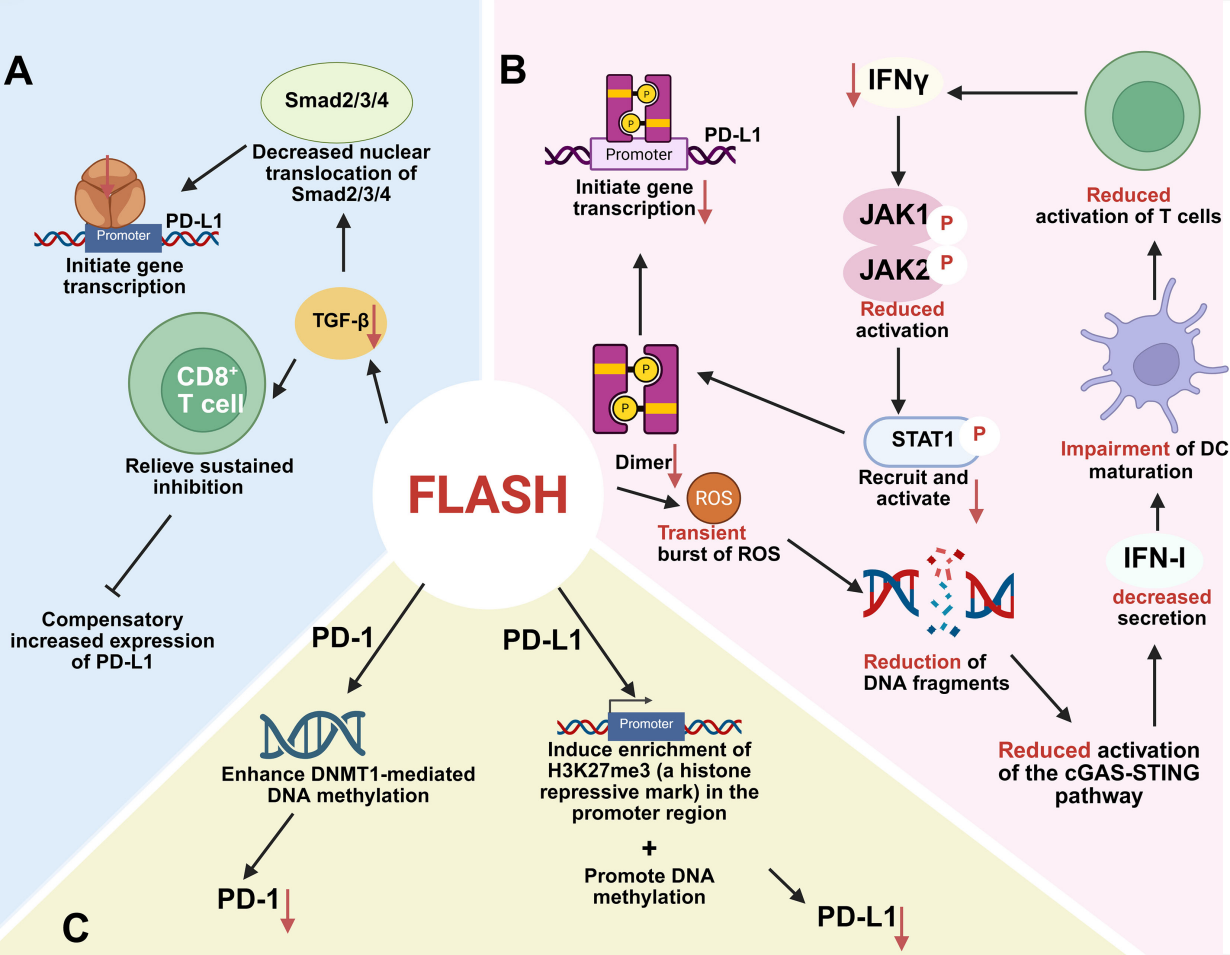
FLASH reduces the expression of PD-L1. **(A)**. FLASH reduces TGF-β secretion, lifting its sustained inhibition of CD8^+^ T cells and decreasing expression of T cell exhaustion markers (e.g., TIM-3). With restored T cell function, the “immune pressure” on the PD-1/PD-L1 pathway decreases, indirectly suppressing compensatory high PD-L1 expression in tumor cells. Simultaneously, FLASH inhibits TGF-β activation by blocking integrin αvβ6, reducing nuclear translocation of the transcription factor Smad3. Weakened Smad3 binding to the PD-L1 promoter directly suppresses PD-L1 expression at the transcriptional level. **(B)** FLASH induces transient oxygen depletion, reducing mitochondrial ROS levels, altering DNA damage patterns, and decreasing cytoplasmic DNA fragmentation. The latter directly inhibits cGAS-STING pathway activation, leading to reduced IFN-I secretion. Reduced IFN-I secretion impairs DC maturation, diminishes antigen presentation efficiency, obstructs CD8^+^ T cell activation, and decreases IFNγ expression. IFNγ activates STAT1 through a series of signaling pathways; STAT1 dimers translocate to the nucleus, bind the PD-L1 promoter, and promote transcription. Thus, reduced IFNγ expression correspondingly decreases PD-L1 expression. **(C)** FLASH induces enrichment of the histone-inactivating mark H3K27me3 in the PD-L1 promoter region while promoting increased DNA methylation. These two epigenetic marks synergistically induce “transcriptional silencing” of the PD-L1 gene, persistently suppressing its expression. In T cells, FLASH enhances DNMT1-mediated DNA methylation, persistently suppressing PD-1 expression. PD-1, as the receptor for PD-L1, exhibits reduced expression that diminishes the ligand-receptor interaction of “PD-1/PD-L1 binding,” indirectly weakening the functional requirement for PD-L1.

If confirmed, epigenetic modulation of PD-L1 could offer a mechanism for more durable suppression than transient signaling pathway inhibition. However, this remains hypothetical, as direct evidence for FLASH-induced epigenetic changes *in vivo* is lacking. This provides an extended temporal window for enhanced efficacy of immunotherapy (125). (**Figure 5**).

However, this persistence is not permanent, and substantial risks of reversal exist during long-term follow-up. These risks stem primarily from three factors: dynamic immune selection pressure and inflammatory signaling changes in the tumor microenvironment; the inherent epigenetic plasticity of tumor cells, which can upregulate relevant demodifying enzymes to erase the repressive marks induced by FLASH; and tumor heterogeneity, which allows the selection and proliferation of cell clones that were not effectively epigenetically reprogrammed. Consequently, the long-term suppression of PD-L1 by FLASH radiotherapy is highly dependent on combination with immunotherapies such as anti-PD-1/PD-L1 inhibitors to sustain immune surveillance and prevent the expansion of resistant clones. Meanwhile, its durability requires validation through long-term clinical follow-up and dynamic monitoring of biomarkers (126). Thus, while these mechanisms are biologically plausible, they currently reside in the realm of hypothesis rather than established fact.

### 3.9 Differential immunological impacts of FLASH radiotherapy fractionation

The demonstrated ability of FLASH radiotherapy to preserve the host’s immune capacity sets the stage for a promising alliance with immunotherapy. However, translating this potential into clinical reality necessitates optimizing the very parameters of radiation delivery to actively steer the immune response. Central to this optimization is the unresolved question of fractionation: does the superior immune preservation conferred by FLASH favor a single, high-intensity exposure to rapidly unleash antigen and danger signals, or a fractionated approach to sustainably prime and expand tumor-specific T-cell clones? Therefore, elucidating the differential effects of single-dose versus hypofractionated FLASH regimens on the dynamics of immune cell recruitment and memory formation is paramount (127). The differences between a single fraction of 15–18 Gy and fractionated doses of 8–10 Gy are presented in the table below (**Table 3**).

**Table 3:** Comparative immunological effects of single-fraction vs. fractionated FLASH radiotherapy.

Fractionation mode	Single fraction (15–18 Gy)	Fractionated (8–10 Gy × 2)
Therapeutic Goal	Maximize immunogenic cell death (ICD) and antigen release; convert “cold” tumors to “hot”.	Balance efficacy and safety; preserve lymphocyte function and sustain immune activation.
Impact on Immune Cell Infiltration	Strong but potentially transient; creates a “cytokine storm” that may recruit immunosuppressive cells (e.g., Tregs) over time.	Sustained and milder; promotes continuous antigen presentation and T-cell recruitment, favoring a more balanced immune microenvironment.
Effect on Immune Memory	High potential for activating broad T-cell responses but risks lymphocyte exhaustion, potentially compromising long-term memory without combinatorial immunotherapy.	Superior for long-term memory formation due to better preservation of circulating lymphocytes and promotion of central memory T-cell (Tcm) differentiation.

While multiple studies have reported FLASH-associated immune changes suggestive of enhanced antitumor immunity—including increased CD8^+^T cell infiltration, reduced Tregs, and M1 macrophage polarization (18)—it is important to recognize that these findings are not universally consistent and may be highly model dependent. For example, some studies show robust immune infiltration after FLASH (18, 105), while others report minimal differences compared to CONV-RT; similarly, reports on cytokine profiles vary across tumor models and radiation modalities (19, 108). Potential sources of discrepancy include differences in radiation dose (low doses may not trigger immune activation), tumor immunogenicity (‘hot’ vs. ‘cold’ tumors), FLASH modality (electron vs. proton), and timing of immune analysis. Critically, no study has directly compared FLASH and CONV-RT across multiple tumor models using identical immunological endpoints, leaving the generalizability of any observed ‘immune advantage’ unknown. Until such systematic comparisons are performed, claims of FLASH-mediated immune enhancement should be interpreted with caution and considered hypothesis-generating rather than established”.

## 4 FLASH combined immunotherapy strategy

### 4.1 The rationale and evidence gaps for combining FLASH-RT with immune checkpoint inhibitors

The combination of FLASH-RT and immune checkpoint inhibitors (ICIs) represents a promising, albeit still experimental, frontier in radio-immunotherapy. This strategy constructs an anti-cancer closed-loop of “immune activation-sustained response” by remodeling the tumor immune microenvironment and relieving immune checkpoint inhibition, thereby providing a new pathway for overcoming refractory tumors. However, clinical studies on FLASH combined with ICIs remain scarce, so its superiority is largely speculative and has certain limitations.

Immune checkpoint inhibitors (ICIs), which target molecules such as PD-1/PD-L1 (to reverse T cell exhaustion) and CTLA-4 (to enhance T cell priming in lymph nodes), act to relieve immune checkpoint suppression. The synergistic logic between the two modalities is clear: FLASH first converts tumors into “hot tumors” characterized by high immune infiltration and low suppression, and ICIs then alleviate residual immunosuppression, thereby enabling durable anti-tumor immunity (128). However, this sequence has not been experimentally validated in a controlled FLASH+ICIs study.

The immune synergistic effect of this combined therapy follows a precisely orchestrated cascade of reactions. FLASH-RT triggers immunogenic cell death (ICD), releasing damage-associated molecular patterns such as HMGB1 and ATP that activate the cGAS–STING pathway and induce a robust surge of typeIinterferons. Meanwhile, FLASH-RT remodels the tumor microenvironment (TME) by reducing immunosuppressive cells, laying the groundwork for immune activation. Tumor antigens and typeIinterferons act in concert to promote dendritic cell (DC) maturation and antigen presentation, which subsequently drives the activation and clonal expansion of CD8^+^ T cells.

Building upon this activated immune state, ICIs inhibit checkpoint molecules such as PD-1 and CTLA-4 (129), enabling effector T cells to overcome tumor-mediated immune suppression, achieve deep tumor infiltration, and ultimately induce long-term immune memory—forming a comprehensive immune cycle.

However, the mechanistic foundation for this synergy is not as straightforward as it may seem, and is complicated by a central, unresolved paradox in FLASH immunobiology: the dual and seemingly opposing modulation of the cGAS-STING pathway. On one hand, FLASH-RT has been shown to reduce cytosolic double-stranded DNA (dsDNA) fragments and dampen the cGAS-STING-driven pro-inflammatory response in normal tissues, which is a key mechanism for its tissue-sparing effect. On the other hand, effective anti-tumor immunity, particularly the priming of T cells by dendritic cells (DCs), is heavily dependent on the activation of the very same cGAS-STING pathway by tumor-derived DNA to produce type I interferons (IFN-I).

This apparent contradiction is likely resolved by considering a dose-dependent threshold for STING activation and the differential context of STING signaling in tumor versus normal tissue. The “FLASH effect” may operate by keeping STING activation below a detrimental, hyper-inflammatory threshold in normal tissues, thereby preventing fibrosis and vascular damage. In contrast, within the tumor microenvironment (TME), the high dose delivered to the tumor, combined with the unique biology of cancer cells (e.g., higher baseline oxidative stress, mitochondrial DNA instability), might still generate sufficient cytosolic DNA to cross the activation threshold, triggering a qualitatively different, immunogenic STING response.

This concept of “immune decoupling” posits that FLASH-RT can spatially separate the detrimental inflammatory consequences of STING activation in normal tissues from its beneficial, immunostimulatory role in the TME. The net effect on the systemic anti-tumor immune response, therefore, depends on a delicate balance. It is not simply that FLASH “activates” or “suppresses” immunity, but rather that it recalibrates the immune system’s response to radiation, minimizing collateral suppression while preserving, or even focusing, the anti-tumor immune assault. This framework has profound implications for combination with ICIs. If FLASH reduces the systemic inflammatory ‘noise’ (e.g., IL-6) that can drive ICIs resistance and toxicity, while maintaining the immunogenic ‘signal’ within the tumor, it could create a more favorable window for ICIs activity. This hypothesis, however, requires direct experimental validation by comparing STING pathway activation and downstream IFN-I signatures in both tumor and distant normal tissues following FLASH versus CONV-RT.

Precise clinical implementation of this combined strategy requires optimized treatment scheduling, dosage selection, and biomarker-guided management. Administration timing is critical: ICIs should be prioritized within 72 hours following FLASH-RT to coincide with the peak of T-cell infiltration, thereby maximizing effector T-cell function through PD-1/CTLA-4 blockade (128). However, this optimal window has not been validated in any FLASH-specific study, and the kinetics of T-cell infiltration after FLASH may differ from CONV-RT due to differences in vascular and immune modulation. It is essential to avoid ICIs administration prior to radiotherapy due to the potential exacerbation of immune-related adverse events (130). Dosage modulation should be tailored to tumor phenotype; immune-cold tumors may benefit from a single fraction of 15–18 Gy (BED ≥ 60 Gy) to maximize antigen release and dendritic cell activation (131); whereas metastatic lesions can be treated with 8–10 Gy in 2 fractions (48-hour interval, BED ≈ 40 Gy) to elicit abscopal effects while preserving lymphocyte function (132). Biomarker assessment further enables personalized management: a post-radiation (72 h) intratumoral CD8^+^/FoxP3^+^ T-cell ratio > 2.0 (133) and serum IFN-β > 50 pg/mL are predictive of superior response (41), whereas plasma IL-6 > 30 pg/mL signifies increased risk of immune-related pneumonitis and warrants prompt intervention (134). However, the predictive thresholds of FLASH radiotherapy-related biomarkers vary significantly across different tumor types, a discrepancy determined by the characteristics of the tumor immune microenvironment and molecular biological properties. For instance, “hot tumors” (e.g., melanoma, some lung cancers) with baseline T cell infiltration often readily meet the CD8^+^/FoxP3^+^ T cell ratio threshold, whereas “cold tumors” (e.g., pancreatic cancer, glioblastoma) require the threshold to be lowered to 1.5 or even lower due to minimal immune infiltration; tumors with high immunosuppression (e.g., liver cancer) may need a higher actual threshold due to elevated baseline levels of Tregs (135). The reference threshold for serum IFN-β also varies with the functionality of the cGAS-STING pathway: tumors with a fully functional cGAS-STING pathway (e.g., some breast cancers) easily meet the threshold, while those with pathway deficiency or inhibition (e.g., some colorectal cancers with STING gene methylation) exhibit persistently low IFN-β levels, diminishing its predictive value—here, levels >20 pg/mL may indicate immune activation, and comprehensive assessment with other biomarkers may be necessary (136). Together, these elements establish a closed-loop framework encompassing precision delivery, response prediction, and toxicity monitoring. It is important to emphasize that all of the above suggestions regarding ICIs timing, biomarker thresholds, and dose selection are hypothesis-generating rather than evidence-based. They are derived from CONV-RT studies and preclinical FLASH observations that have not yet been validated in human trials. The translational maturity of FLASH-ICIs combinations remains extremely low, and clinical decision-making must await dedicated Phase I/II trials that incorporate immune monitoring from the outset. Readers should interpret these concepts as directions for future research, not as guidelines for current practice.

The combined therapy must carefully balance efficacy and safety while addressing challenges related to toxicity and resistance. In terms of toxicity, FLASH-RT may potentially exacerbate pulmonary or intestinal toxicity associated with ICIs—particularly when combined with CTLA-4 inhibitors (5) Mitigation strategies include the use of fractionated FLASH regimens (e.g., 10 Gy × 2 fractions separated by a 48-hour interval) to reduce biological impact, or co-administration with IL-1β antagonists such as anakinra to suppress excessive inflammation (137). With regard to therapeutic resistance, residual tumors following irradiation often demonstrate upregulation of alternative immune checkpoints including TIM-3 and LAG-3 (138). This resistance and recurrence is a dynamically evolving process driven by interactions between FLASH-RT’s unique biological effects and the tumor microenvironment (TME). It involves differential cGAS-STING pathway regulation and immune pressure diversion: FLASH-RT’s ultra-high dose rate reduces cytoplasmic DNA fragments, potentially abrogating excessive cGAS-STING activation. While protecting normal tissues and mitigating inflammation, this modulates TME immune alert signals. After eliminating PD-L1-high clones, antigens released via immunogenic cell death (ICD) impose strong immune selection pressure; insufficient cGAS-STING-type I interferon axis responses enrich innate or adaptive TIM-3/LAG-3-dependent (rather than PD-1/PD-L1-dependent) clones. Moreover, FLASH-RT exerts complex effects on tumor hypoxia and HIF-1α: while alleviating hypoxia may inhibit HIF-1α-driven checkpoint expression, incomplete hypoxic improvement allows residual cells to sustain HIF-1α-dependent TIM-3/LAG-3 upregulation (139). A sequential triple-blockade approach combining anti-PD-1, anti-TIM-3, and anti-LAG-3 antibodies may be necessary to simultaneously target multiple escape pathways, thereby progressively limiting opportunities for tumor immune evasion.

Beyond biological uncertainties, the translational feasibility of FLASH-ICIs combinations is constrained by modality-specific technical barriers. Beam modality dictates target accessibility: electron FLASH is clinically available but limited to superficial tumors, while proton FLASH is required for deep-seated lesions but faces a trade-off between UHDR maintenance and dosimetric conformality—pencil beam scanning sacrifices UHDR across the target volume, whereas transmission beams achieve UHDR but lose the Bragg peak advantage. Pulse structure parameters (instantaneous dose rate, pulse width, repetition frequency) vary widely across accelerators and may independently influence immune responses, yet systematic comparisons are lacking. Field size constraints limit current FLASH delivery to small volumes; treating large or multiple tumors—common in ICIs candidates—is not yet feasible. Treatment planning for FLASH remains primitive, with optimization algorithms for UHDR coverage still investigational. *In vivo* dosimetry under UHDR is unavailable, meaning delivered doses cannot be verified in real time—a major obstacle for dose-finding ICIs trials. Finally, partial-volume irradiation—inevitable with current small fields—may alter antigen release kinetics and immune cell trafficking in unpredictable ways that differ from whole-tumor irradiation. Collectively, these barriers underscore that FLASH-ICIs combinations are not a single strategy but a family of possibilities, each constrained by specific technical limits. Until these engineering challenges are resolved, clinical translation will remain confined to superficial targets.

### 4.2 FLASH-RT and CAR-T cell therapy – promise and barriers in solid tumors

As a milestone in precision cancer therapy, chimeric antigen receptor T-cell (CAR-T) therapy involves genetically engineering patients’ autologous T cells to express chimeric antigen receptors (CARs). These synthetic receptors typically consist of an extracellular antigen-binding domain—often derived from an antibody single-chain variable fragment (scFv)—a transmembrane domain, and an intracellular signaling domain comprising the CD3ζ chain coupled with costimulatory molecules such as CD28 or 4-1BB (140). This design enables CAR-T cells to recognize tumor antigens in a non-MHC-restricted manner, thereby facilitating precise and potent antitumor activity.

The therapeutic process follows a standardized pathway: blood collection → T cell isolation → CAR gene transduction → ex vivo expansion → reinfusion into the patient. Following genetic modification, CAR-T cells become strongly activated and undergo sustained expansion upon antigen engagement, functioning as “living drugs” that mediate persistent anti-tumor activity. In hematological malignancies, CAR-T therapy demonstrates several key advantages, including highly specific targeting (e.g., against CD19 or BCMA), efficacy against refractory or relapsed disease, and long-term persistence due to the formation of memory T cells (141, 142).

However, multiple limitations continue to restrict further breakthroughs in CAR-T therapy. These include poor efficacy in solid tumors—due to the immunosuppressive microenvironment, antigen heterogeneity, and inadequate T-cell infiltration—as well as severe toxicities such as cytokine release syndrome (CRS) and immune effector cell-associated neurotoxicity syndrome (ICANS). Additional challenges comprise myelosuppression and infections associated with lymphodepleting chemotherapy, antigen escape, and high treatment costs (143–146).

FLASH has been proposed as a potential strategy to address some limitations of CAR-T therapy. Furthermore, its capacity to release tumor antigens and modulate the immune microenvironment (131) provides a rational foundation for synergistic combination strategies.

The core rationale for combining these modalities lies in the ability of FLASH-RT to complement CAR-T therapy across three key dimensions: enhancing efficacy in solid tumors, reducing pretreatment toxicity, and reinforcing antitumor immunity.

FLASH promotes immunogenic cell death of tumors and remodels the tumor immune microenvironment. It remodels tumor vasculature to improve permeability, thereby enhancing CAR-T cell infiltration (21). Moreover, its nonspecific cytolytic action helps overcome antigenic heterogeneity by releasing diverse antigens, thereby addressing a major limitation of CAR-T therapy in solid tumors (147). Furthermore, FLASH efficiently depletes host lymphocytes, creating space for adoptive CAR-T cell engraftment and expansion, while its normal tissue-sparing properties reduce the risk of bone marrow and gastrointestinal damage, indirectly mitigating the severity of CRS and ICANS. The immunogenic cell death (ICD) induced by FLASH activates dendritic cells and boosts endogenous T-cell responses, establishing a coordinated “two-pronged” attack alongside CAR-T cells. Notably, localized FLASH irradiation may even disrupt physical barriers such as the blood–brain barrier, potentially enabling CAR-T cells to target refractory intracranial malignancies.

A study by Ni et al. published in *Nature Cancer* demonstrated that FLASH-RT specifically reprograms tumor lipid metabolism in medulloblastoma, significantly reducing the proportion of immunosuppressive tumor-associated macrophages (TAMs) while simultaneously enhancing CD8^+^ T cell infiltration. The combination of FLASH and CAR-T cell therapy resulted in a 70% higher survival rate in mice compared to monotherapy groups. In contrast to CONV-RT, FLASH, at equivalent doses, preserves hippocampal neural stem cells, mitigates neurotoxicity commonly associated with CAR-T therapy, and reduces myelosuppression risks, thereby establishing a safety window for combinatorial treatment (148).

CAR-T therapy for solid tumors often targets deep-seated or intracranial lesions (e.g., glioblastoma). While electron FLASH is suitable for superficial CAR-T targets (e.g., melanoma skin metastases), proton or heavy ion FLASH would be required for intracranial delivery. However, no clinical data exist for proton FLASH combined with CAR-T, and the technical challenges of maintaining UHDR in the brain while sparing critical structures (e.g., hippocampus) are formidable. Currently, FLASH-CAR-T combinations remain a conceptual rather than clinically actionable strategy.

Despite its promising prospects, the combination of FLASH-RT and CAR-T therapy faces several scientific and clinical challenges that must be resolved before widespread clinical translation. The biological mechanisms underlying FLASH-induced protection of normal tissues and its specific immunomodulatory effects on the tumor microenvironment remain incompletely elucidated. Furthermore, ultra-high dose rate radiotherapy equipment remains scarce, and parameters such as the optimal timing, sequencing, and dosing for combining irradiation with CAR-T infusion require systematic investigation. Large-scale clinical data are still lacking, particularly regarding patient selection (e.g., suitable tumor types and target antigens), long-term safety of FLASH total body irradiation (TBI), and risks of cytokine release syndrome (CRS) or immune effector cell-associated neurotoxicity syndrome (ICANS) following combination therapy. The absence of validated molecular biomarkers for predicting treatment efficacy further impedes clinical progress.

Nevertheless, the core objective of combining FLASH with CAR-T therapy—overcoming the efficacy limitations of CAR-T in solid tumors while reducing the toxicity associated with conventional preconditioning regimens—has firmly established this strategy as an innovative direction in cancer treatment. FLASH serves a dual role: as a “pioneer for solid tumors” by remodeling the tumor microenvironment and enhancing T-cell infiltration via localized irradiation, and as a “toxicity reducer” by potentially replacing conventional lymphodepleting chemotherapy with FLASH total body irradiation (TBI). Moving forward, efforts should focus on accelerating the clinical adoption of FLASH-compatible equipment, conducting well-designed head-to-head clinical trials (e.g., comparing FLASH-TBI with traditional lymphodepletion), and exploring triple-combination strategies incorporating immune checkpoint inhibitors and other immunomodulators. Although this combined approach remains in its early stages, it represents a conceptually promising direction that may, if validated, contribute to overcoming solid tumors and refining the treatment of hematologic malignancies. Whether it ultimately alters clinical practice will depend on rigorous prospective testing.

### 4.3 FLASH-RT combined with oncolytic viruses or cancer vaccines – synergy still theoretical

The combination of FLASH-RT with oncolytic viruses or cancer vaccines has been proposed as a potentially synergistic strategy in oncology. It seeks to synergize the strengths of distinct treatment modalities, overcome the limitations inherent to individual approaches, and establish a coordinated anti-cancer system characterized by “local killing–immune activation–systemic protection”. However, such advantages are largely based on speculation, with limited clinical data available. Therefore, this approach still has certain inaccuracies and limitations.

FLASH-RT establishes an immunologically favorable microenvironment, thereby laying a critical foundation for multimodal combination strategies. Oncolytic viruses and cancer vaccines activate antitumor immunity through distinct yet complementary mechanisms. Oncolytic viruses selectively infect and lyse tumor cells, releasing danger signals during replication and cell rupture that potently activate both innate and adaptive immune responses. They can additionally reverse the immunosuppressive tumor microenvironment by reducing regulatory T cells (Tregs) and repolarizing tumor-associated macrophages. Through genetic engineering, these viruses can be armed with immunostimulatory cytokines such as GM-CSF and IL-12, or engineered to express immune checkpoint inhibitors, further enhancing their immunotherapeutic potency (149). Cancer vaccines, conversely, focus on active immune priming. They deliver tumor-specific antigens—including neoantigens and shared tumor antigens—in combination with adjuvants to directly activate and expand tumor-specific T cells. This approach induces durable immune memory, thereby providing long-term protection against recurrence and establishing a sustained defensive barrier against cancer (150).

These three modalities operate through a multi-node complementary logic to achieve synergistic antitumor effects. At the antigen level, FLASH efficiently kills tumor cells to release a broad spectrum of antigens, while oncolytic viruses further amplify antigen exposure through their lytic activity (149), and cancer vaccines enable precise delivery of selected target antigens (150); together, they maximize tumor antigen presentation and enhance T cell recognition. Within the tumor microenvironment (TME), FLASH alleviates radiotherapy-induced immunosuppression, oncolytic viruses directly disrupt the suppressive TME (149), and cancer vaccines provide potent T cell activation signals (150), collectively converting immunologically “cold” tumors into “hot” ones. In terms of spatiotemporal dynamics, FLASH rapidly debulks tumors to create a favorable setting for viral and vaccine activity, oncolytic viruses infect and eliminate radiation-resistant residual tumor cells (149), and cancer vaccines amplify and sustain systemic immune responses (150), thereby establishing a therapeutic closed-loop of tumor reduction, residual clearance, and immune memory. Furthermore, the normal tissue-sparing advantage of FLASH, combined with the manageable toxicity profile of oncolytic viruses and the generally favorable safety of cancer vaccines, is expected to enhance therapeutic efficacy while minimizing the severe adverse effects commonly associated with traditional combination regimens.

Oncolytic virus trials often involve intratumoral injection into accessible lesions (e.g., melanoma, head and neck cancer). Electron FLASH could theoretically be applied to the same superficial tumors, but the optimal sequencing (virus before or after FLASH) and the impact of FLASH on viral replication are unknown. For visceral tumors requiring proton FLASH, the additional complexity of beam delivery and partial-volume irradiation effects on virus distribution have not been studied.

In clinical translation, treatment sequencing represents a key area for exploration. Current research favors a “FLASH-first” strategy, wherein its capacity for rapid tumor debulking, antigen release, and remodeling of the tumor microenvironment (TME) may create a critical window of opportunity for subsequent oncolytic virus replication and dissemination, as well as vaccine-primed T-cell infiltration. The peak of post-radiation inflammation (occurring within hours to days after treatment) may represent the optimal timing for vaccination. Alternatively, a “virus/vaccine-first” model—pre-sensitizing the immune system—could potentially potentiate the immunogenic effects of FLASH; however, such synchronous or alternating regimens require careful evaluation of their biological interactions and potential synergistic or inhibitory effects.

The selection of oncolytic viruses should prioritize those with inherent tumor tropism, such as herpes simplex virus type 1 (HSV-1) and adenoviruses. Further genetic engineering to enable the expression of immunostimulatory cytokines or immune checkpoint-blocking antibodies can significantly enhance synergistic efficacy. Meanwhile, the design of cancer vaccines requires a balanced approach between personalized neoantigens—which entail higher costs and lengthier development timelines—and shared tumor antigens. Alternatively, vaccine strategies could leverage antigens released *in situ* by oncolytic virotherapy or FLASH-RT.

Regarding clinical indications, priority should be given to: (1) radiosensitive solid tumors exhibiting moderate-to-low immunogenicity, such as lung cancer, glioblastoma, and melanoma; and (2) locally advanced or oligometastatic disease. The former context benefits from the combined strategy to overcome immunological resistance, while the latter capitalizes on the synergy between potent local tumor control and systemic immune activation—achieving the dual objectives of primary site eradication and inhibition of distant metastases.

This combined strategy precisely connects the anti-cancer links of “local killing, immune activation, and memory maintenance”. Although it needs to address challenges such as timing optimization, viral engineering, and vaccine personalization, its potential for efficacy enhancement and toxicity reduction has already illuminated new possibilities for the future of tumor treatment—shifting from “single-modal attack” to “systematic anti-cancer therapy”, truly achieving dual breakthroughs in efficacy and safety, and paving a brand-new path for overcoming refractory tumors.

### 4.4 High-Z nanoparticles with FLASH-RT – radiosensitization or disruption of the FLASH effect

The integration of high-atomic-number (high-Z) metal nano-reagents with FLASH radiotherapy has been explored as a potential strategy to enhance local tumor dose deposition. This combination necessitates a nuanced examination of their interaction, particularly concerning the fundamental parameters of the FLASH effect: the dose-rate threshold​ for normal tissue sparing and the resultant immunomodulatory landscape (151).

The core mechanism of high-Z nano-reagents is the enhancement of local energy deposition via photoelectric absorption and Compton scattering, leading to a cascade of low-energy secondary electrons. This phenomenon intersects critically with the proposed physicochemical mechanisms of FLASH (151).

The localized dose amplification within the tumor could potentially lower the effective external radiation dose required to achieve a therapeutic tumor response under FLASH conditions. If the FLASH protective effect is contingent on a specific, absolute dose threshold in normal tissue, achieving equivalent tumor cell kill with a lower incident dose due to nanoparticle radiosensitization would be highly advantageous. This is conceptually aligned with the oxygen depletion hypothesis; the intense, nanoparticle-driven radiolysis could create an ultra-transient hypoxic microenvironment at the nanoscale, potentially amplifying the FLASH-specific radioprotective signaling in adjacent normal tissues. Monte Carlo (MC) simulations using the Geant4-DNA code have provided a computational basis for this concept, demonstrating that gold nanoparticles (GNPs) of varying sizes can significantly enhance the chemical yield of reactive oxygen species (ROS) near their surface when irradiated with ultrahigh dose rate (UHDR) electron beams. This localized ROS “boost,” quantified by a yield enhancement factor (YEF), was shown to be particularly pronounced with smaller GNP sizes and at lower UHDR settings, intensifying DNA damage, especially double-strand breaks, within critical biomolecular targets (151).

Conversely, the massive, localized generation of free radicals by nanoparticles risks disrupting the precise spatiotemporal dynamics believed to be essential for the FLASH effect. The FLASH effect may rely on a specific, transient balance between radical species (e.g., the ratio of •OH to O_2_•^−^). The nanoparticle-induced radical “storm” could overwhelm the delicate recombination kinetics that underpin normal tissue protection in FLASH-RT. Furthermore, non-specific uptake of nanoparticles in normal tissues could lead to localized “hot spots” of dose amplification, potentially eroding the very tissue-sparing advantage that defines FLASH. This risk is underscored by the fact that the enhanced ROS production, while therapeutically beneficial for tumor control, could directly damage infiltrating immune cells or the antigen-presentation machinery within the tumor microenvironment if not precisely confined (152).

The combination holds significant but complex promise for activating anti-tumor immunity. Beyond gold nanoparticles, novel self-assembled nanoparticles containing dysprosium and manganese ions (Dy/Mn-P NPs) have been developed to act not only as radiosensitizers but also as potent immune modulators. These Dy/Mn-P NPs have demonstrated the ability to improve the tumor microenvironment and significantly increase endogenous ROS levels within the tumor. Crucially, this enhanced ROS generation was shown to activate the stimulator of interferon genes (STING) pathway, an innate immune signaling cascade, leading to the secretion of pro-inflammatory immune factors and the maturation of dendritic cells (DCs), thereby triggering a robust anti-tumor immune response (152).

The combination of FLASH-RT with high-Z nanoparticles holds significant synergistic potential. High-Z nanoparticles can dramatically enhance immunogenic cell death within the tumor, leading to a greater release of tumor-associated antigens and damage-associated molecular patterns (DAMPs). This “*in situ* vaccination” effect can synergize powerfully with FLASH-RT’s unique ability to preserve the host’s immune competence. By sparing circulating lymphocytes and reducing immunosuppressive cytokine production (e.g., TGF-β), FLASH-RT creates a favorable systemic environment for the nanoparticles’ locally enhanced antigen release to prime a robust, *de novo* T-cell response. The Dy/Mn-P NPs exemplify this potential, as their ability to activate the STING pathway during radiotherapy can directly amplify this immunostimulatory cascade (152).

However, the “explosive” oxidative damage from nanoparticles is a double-edged sword. While boosting tumor cell killing, it may drive non-immunogenic cell death (e.g., necrosis) instead of immunogenic apoptosis. Altered antigen release kinetics—too rapid or chaotic—can hinder dendritic cell cross-presentation, and widespread radical generation may damage infiltrating immune cells or antigen-presentation machinery in the tumor microenvironment. Critically, nanoparticle-modified radical distribution could interfere with the immunomodulatory signals (e.g., reduced peroxynitrite stress) that underpin FLASH-induced lymphocyte sparing. This interplay creates uncertainty, as the net immunostimulatory effect hinges on a delicate balance between enhanced antigen availability and a functional immune ecosystem, which nanoparticles may disrupt. Monte Carlo simulations further emphasize this complexity, showing the net effect depends on precise parameters (nanoparticle size, beam energy, dose rate) that require meticulous optimization to favor therapeutic benefit (151).

High-Z nanoparticle studies are almost exclusively preclinical and use electron or photon FLASH. Translating this combination to deep tumors would require proton FLASH, but the interaction of nanoparticles with proton beams under UHDR conditions is unexplored.

**Figure 6:**
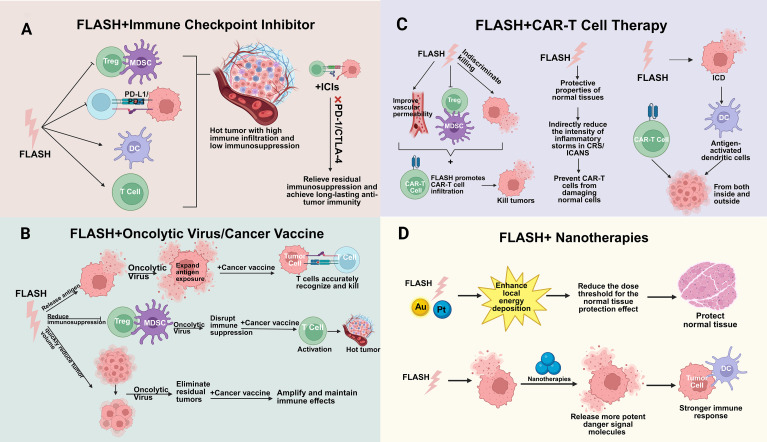
FLASH Combined Immunotherapy Strategy. **(A)** When combined with immune checkpoint inhibitors (ICIs), FLASH can reshape the tumor microenvironment by reducing immune-suppressive cells such as regulatory T cells (Tregs) and myeloid-derived suppressor cells (MDSCs), modulating molecules like PD-L1, and transforming tumors into “hot tumors” rich in immune infiltration and with weakened immunosuppression. This myeloid-derived suppressor cells (MDSCs), and modulate molecules like PD-L1. This transforms tumors into “hot tumors” characterized by abundant immune infiltration and reduced immunosuppression. When combined with ICIs such as anti-PD-1/CTLA-4, residual immunosuppression is alleviated, enabling sustained antitumor immunity. **(B)** When combined with oncolytic viruses/cancer vaccines, FLASH releases tumor antigens, reduces immunosuppression, and shrinks tumor volume. Oncolytic viruses enhance antigen exposure, break immune suppression, and eliminate residual tumors. When integrated with cancer vaccines, this combination enables T cells to precisely recognize and kill tumor cells while activating them to transform tumors into “hot tumors”. It also amplifies and sustains immune responses. **(C)** When combined with CAR-T cell therapy, FLASH non-specifically kills tumors while improving vascular permeability to enhance CAR-T cell infiltration and tumor lysis. Its protective effect on normal tissues indirectly reduces the inflammatory intensity of cytokine release syndrome (CRS) or immune effector cell-associated neurotoxicity syndrome (ICANS), preventing CAR-T cells from damaging healthy cells. Simultaneously, FLASH induces immunogenic cell death (ICD) in tumor cells, activating antigen-presenting dendritic cells (DCs). This facilitates CAR-T cell recognition and tumor killing from both inside and outside the tumor. **(D)** When combined with nanotherapies, FLASH exhibits dual beneficial effects. First, when paired with nanomaterials such as gold (Au) or platinum (Pt), FLASH enhances local energy deposition within tumors. This reduces the dose threshold required to achieve the FLASH-mediated normal tissue protection effect, thereby safeguarding healthy tissues more effectively. Second, the combination of FLASH with nanotherapies amplifies the release of more potent danger signal molecules. This robustly activates dendritic cells (DCs), leading to a stronger and more sustained antitumor immune response.

In summary, the combination of FLASH-RT with high-Z nano-reagents is not merely additive but represents a complex interplay that can be either synergistic or antagonistic. The outcome hinges on achieving highly selective tumor targeting​ of the nanoparticles to harness their dose-amplifying and immunostimulatory potential within the malignancy while absolutely minimizing their presence in surrounding normal tissues​ to preserve the integrity of the FLASH effect. Future research must focus on engineering smart, targetable nanoparticles and meticulously mapping the physicochemical and biological cascades they initiate under UHDR irradiation to safely unlock the full potential of this powerful combination therapy (**Figure 6**).

### 4.5 Comparative perspectives: FLASH versus low-dose radiotherapy in immuno-oncology

Having explored the potential of FLASH radiotherapy to synergize with immunotherapy, it is instructive to contextualize this approach within the broader landscape of radio-immunotherapy, particularly in comparison to the established paradigm of low-dose radiotherapy.

Low-Dose Radiotherapy (LDRT) and FLASH Radiotherapy (FLASH-RT) are not competing modalities but complementary radio-immunotherapeutic approaches, each serving distinct clinical niches. LDRT is characterized by low-toxicity modulation of the tumor immune microenvironment and can further exert synergistic effects by reprogramming the tumor microenvironment—such as normalizing tumor vasculature to improve drug delivery and converting immunosuppressive M2 macrophages to immunostimulatory M1 phenotypes—thereby priming the tumor for subsequent therapies. It is well-suited for treating metastatic disease or acting as an immune primer, though its tumoricidal efficacy as a monotherapy is limited (25, 153). In contrast, FLASH-RT transcends the traditional trade-off between dose rate and therapeutic efficacy through its ultra-high dose rate, enabling potent tumor cell killing at high doses while sparing normal tissues, efficiently inducing immunogenic cell death and antigen release, and activating and amplifying anti-tumor immune responses. Moving forward, the strategic selection or sequential administration of these two modalities should be tailored to tumor type and immune context: LDRT for systemic immune conditioning, and FLASH-RT for aggressive local control and robust immune initiation, particularly in combination with immunotherapy (154).

## 5 Clinical translation of FLASH-RT current status and persistent obstacles

Having outlined the diverse advantages of FLASH-RT, the next critical step lies in translating these preclinical promises into real-world clinical benefit. The translation of FLASH radiotherapy from benchtop to bedside is often portrayed in the literature as a linear trajectory of accelerating success. A more critical examination, however, reveals a complex and uneven landscape marked by encouraging proof-of-concept data alongside inconsistent preclinical findings, unresolved technical bottlenecks, and a near-complete absence of efficacy-focused human trials. This chapter centers on FLASH’s clinical translation journey: it first reviews animal experiments that established its foundational safety and efficacy, then summarizes insights from early-stage clinical research in human cohorts, and finally addresses key clinical translation bottlenecks impeding its broader application. Together, these sections map the path from laboratory potential to clinical practice for this innovative radiotherapy modality.

### 5.1 Reproducible findings and inconsistent observations in preclinical FLASH studies

Current preclinical studies on FLASH-RT are primarily conducted using *in vitro* cellular models and *in vivo* animal models, employing radiation sources such as electrons, photons, or protons. The majority of these studies have demonstrated that, compared to CONV-RT, FLASH-RT effectively protects normal tissues and reduces radiation-induced toxicity while maintaining comparable tumor-killing efficacy. A summary of some of the latest preclinical models is provided below (**Table 4**).

**Table 4:** Recent preclinical studies on FLASH radiotherapy.

Author	Year	Animal model	Tumor type	Irradiation particle	Total dose (Gy)	Conventional dose rate (Gy/s)	FLASH dose rate (Gy/s)	Biological effects of FLASH on normal tissues	Immune-related indicator changes
Liljedahl et al. (165)	2022	Mouse (Brain)	NSI glioblastoma	Electron	25	0.13	0.5×10^6^	Protects normal tissue	Reduce the infiltration of immunosuppressive cells and increase the infiltration of cytotoxic CD8^+^ T cells in tumors
Eggold et al. (126)	2022	Mouse (Brain)	Ovarian cancer	Electron	14	0.126	210	Reduces intestinal adverse reactions and enhances immune efficacy	Enhanced efficacy of immune checkpoint inhibitors (anti-PD-1)
Zhu et al. (166)	2022	Mouse (Brain)	Normal tissue	Photon	15	0.1	>150	Protects intestinal tissue and function	Protecting intestinal immune function
Sorensen et al. (167)	2022	Mouse (Lower limb)	Breast cancer xenograft	Proton	60	0.38	83	Reduces acute skin reactions and fibrosis	
Rohrer Bley et al. (168)	2022	Cat (Nose)	Nasal squamous cell carcinoma	Electron	30,48	0.1	6.3×10^6^	Reduces acute radiation damage to skin and bone	
Iturri et al. (169)	2023	Mouse (Brain)	Glioblastoma	Proton	25	4	257	Significant protective effect on the mouse memory center	
Limoli et al. (170)	2023	Mouse (Brain)	Normal tissue	Electron	30	0.09	1.6×10^6^	Protects synaptic plasticity in the mouse hippocampus	
Alaghband et al. (171)	2023	Mouse (Brain)	Normal tissue	Electron	30	0.09	5.6×10^6^	Protects the mouse nervous system	
Dai et al. (172)	2023	Mouse (Brain)	Lung cancer xenograft	Proton	20	0.033	200	Reduces the occurrence of acute radiation pneumonitis	Alleviate pathological immune inflammatory responses in the lungs
Shukla et al. (18)	2023	Mouse (Brain)	Orthotopic Lewis lung cancer	Proton	18	0.05	60	Alters the immune microenvironment and modulates the immune system	Enhance the infiltration of CD8^+^ T cells within tumors; promote the polarization of macrophages toward the anti-tumor M1 phenotype; downregulate the expression of the immunosuppressive molecule PD-L1.
Zhang et al. (173)	2023	Mouse (Skin)	Normal tissue	Proton	45	0.4	130	Protective effect on the skin	
Ghannam et al. (174)	2023	Zebrafish (Embryo)	Normal tissue	Helium ion	24	0.25	9.7×10^6^	Protective effect on the embryo	

Evidence from animal experiments—encompassing multi-organ protection, preservation of antitumor efficacy, and validation in large animals—has demonstrated reproducible FLASH effects in several models, but also revealed substantial inter-model variability and unresolved tissue-specific failures.

Regarding the combination of FLASH-RT and immunotherapy, it is important to note that clinical data for this treatment modality remain exceedingly limited. A relevant preclinical study was conducted by Eggold et al. in 2022 (126), which investigated the potential of abdominopelvic FLASH-RT combined with PD-1 inhibition. The authors demonstrated in an ovarian cancer mouse model that FLASH-RT promoted intestinal regeneration while maintaining tumor control (126). Specifically, the treatment reduced tumor burden by decreasing intratumoral regulatory T cells (Tregs) and increasing cytotoxic CD8^+^ T cell infiltration. Compared to CONV-RT, FLASH not only enhanced T cell infiltration into tumors but also preserved the capacity to improve anti-PD-1 efficacy (126). These findings suggest that FLASH-RT, when combined with immune checkpoint inhibitors (ICIs) therapy, may represent a safer and more effective strategy—reducing radiation-induced toxicity while retaining immunomodulatory potency.

In a study on FLASH-RT for medulloblastoma published by Ni et al. (148) in *Nature Cancer*, it was demonstrated for the first time that FLASH can overcome physical barriers (such as dense stroma) within solid tumors that limit CAR-T cell infiltration. Through remodeling the metabolic-immune axis, FLASH converted immunologically “cold” tumors into “hot” tumors. These findings provide important insights into the potential synergy between FLASH-RT and immunotherapy.

Despite the largely positive tone of preclinical FLASH literature, several inconsistent and negative findings warrant explicit acknowledgment. No FLASH protective effect was detected in mouse gonads in a study specifically designed to test gonadal sparing—this is not a statistically underpowered null result (155), but direct evidence that the FLASH effect is not universal, challenging any proposed single, tissue-independent mechanism.

Moreover, key veterinary studies show substantial variability in outcomes: the widely cited large-animal study in mini-pigs and cats by Vozenin et al. demonstrated highly heterogeneous tumor responses, confirming that FLASH does not guarantee superior tumor control. The field also suffers from publication bias, as negative findings due to suboptimal parameters or biological non-responsiveness are rarely published, creating an inflated impression of universal efficacy.

### 5.2 Feasibility without proven efficacy in early FLASH-RT clinical trials

Initial clinical feasibility has been demonstrated in human trials of FLASH-RT for superficial targets (e.g., bone metastases, skin cancer) (10, 156, 157). These studies are dose-finding and safety trials; they do not provide efficacy data or comparisons with CONV-RT for oncologic outcomes. Among completed trials, the FAST-01 trial—launched in 2021 by the Proton Center at Cincinnati Children’s Hospital Medical Center—enrolled ten patients with painful bone metastases. Using proton FLASH-RT at a dose rate of ≥40 Gy/s and a single fraction of 8 Gy, this study provided the first human data: pain relief rates were comparable to those of CONV-RT, with no observed acute toxicities such as skin injury or bone marrow damage (10).

Building on these findings, the same institution initiated the FAST-02 trial in 2022, which focuses on proton FLASH-RT for thoracic bone metastases. Ten patients received 8 Gy FLASH irradiation, and the trial results are anticipated to be published in 2027 (156).

Additionally, the IMPULSE trial conducted at the University Hospital of Lausanne (Switzerland) is a Phase I dose-escalation study (NCT04986696). It employs 9 MeV electron beams to deliver FLASH-RT to patients with cutaneous melanoma metastases, administering a total dose ranging from 22 to 34 Gy. The primary objective is to determine the maximum tolerated dose (MTD), defined as the dose at which acute skin reactions remain below Grade 3. Secondary endpoints include the evaluation of adverse events and assessment of antitumor efficacy over a 12-month follow-up period (157).

In 2024, the University of Zurich initiated the Flash-Skin I trial (NCT06266836), which enrolled 10 adult and elderly patients with metastatic melanoma. This study utilizes electron-based FLASH-RT with the aim of evaluating the feasibility and safety of this approach for treating cutaneous melanoma metastases. Feasibility is defined as achieving radiotherapy dose accuracy within ±10% per fraction, while the safety criterion specifies that no more than two out of six patients experience dose-limiting toxicity (DLT). The primary endpoints are the incidence of DLT and dose delivery accuracy within 9 months; secondary endpoints encompass pain relief, control of bleeding within 21 months, local tumor response assessment within 9 months, and long-term side effects within 21 months. The results of this trial have not yet been published.

The LANCE trial (NCT05724875), conducted by the Centre Hospitalier Universitaire Vaudois, is currently recruiting participants. The trial plans to enroll 60 adult and elderly patients diagnosed with localized cutaneous squamous cell carcinoma (cSCC) or basal cell carcinoma (BCC). This single-center, randomized Phase II study aims to compare the therapeutic efficacy of FLASH-RT versus CONV-RT for skin cancer. Primary endpoints include the incidence of Grade ≥3 dermatologic adverse events within 6 weeks post-radiotherapy and the local control rate at 12 months. Secondary endpoints encompass multiple aspects, such as the frequency of acute side effects within 3 months after treatment, late side effects occurring between 3 and 12 months, tumor response evaluation, normal tissue reaction assessment, and comparative analysis of various skin parameters—including epidermal thickness, roughness, and vascular den In the future, with the clinical adaptation and widespread adoption of FLASH-RT equipment—such as upgraded electron linear accelerators and advances in proton FLASH technology—coupled with progress in multi-center clinical studies, this technology is poised to reshape the landscape of radiation oncology. It promises to transition radiotherapy from the traditional paradigm of “sacrificing normal tissues to achieve tumor control” to a new era of precision radiotherapy characterized by “normal tissue protection and enhanced anti-tumor immunity.” This evolution will offer patients treatment options marked by higher efficacy, reduced toxicity, and improved survival, ultimately enabling a revolutionary shift from invasive irradiation toward precision protective therapy (**Table 5**).

**Table 5:** FLASH radiotherapy clinical trial.

NCT number	Study title	Anthor	Enrollment	Conditions	Treatment dose	Purpose	Phase	Results
NCT05524064	FLASH Radiotherapy for the Treatment of Symptomatic Bone Metastases in the Thorax	Varian, a Siemens Healthineers Company	10	Bone Metastases in the Thorax	8 Gy/1 fraction. (Dose rate ≥ 40 Gy/s)	Assess toxicities of FLASH radiotherapy and pain relief in subjects with painful thoracic bone metastases	Active, not recruiting.	No results posted
NCT04592887	Feasibility Study of FLASH Radiotherapy for the Treatment of Symptomatic Bone Metastases	Varian, a Siemens Healthineers Company	10	Bone Metastasis	8 Gy/1 fraction. (Dose rate ≥ 40 Gy/s)	Assess feasibility of FLASH radiotherapy for palliative treatment of painful bone metastases	Completed.	Short treatment time, low toxicity (11 grade 1 and 1 grade 2 adverse events)
NCT04986696	Irradiation of Melanoma in a Pulse	Centre Hospitalier Universitaire Vaudois	46	Metastasis From Malignant Melanoma of Skin	FLASH therapy (single dose escalation using Mobetron^®^ with HDR functionality)	Evaluate dose escalation of FLASH radiotherapy as single dose treatment for skin melanoma metastases	Recruiting - Phase 1	No results posted
NCT05724875	FLASH Radiotherapy for Skin Cancer	Centre Hospitalier Universitaire Vaudois	60	Basal Cell Carcinoma Cutaneous Squamous Cell Carcinoma	FLASH RT vs Conventional RT (Dose and fractionation individualized per standard guidelines)	Compare toxicity and efficacy of FLASH radiotherapy versus standard radiotherapy in localized cSCC or BCC	Recruiting - Phase 2	No results posted
NCT06549439	eFLASH for Skin Lesions of Malignant Melanomas	University of Zurich	10	Metastatic Melanoma	Electron FLASH radiotherapy: 15-20 Gy total in 3-5 fractions.(Dose rate ≥ 40 Gy/s)	Assess feasibility and safety of electron FLASH radiotherapy for treatment of melanoma skin metastases	Active, not recruiting - Phase 1.	No results posted

Early clinical trials have verified the feasibility and preliminary safety of FLASH radiotherapy in humans, marking a crucial step toward its clinical translation. However, their limitations are notable: small scale, single objectives (e.g., pain relief), and the lack of exploration into combination with systemic therapies such as immune checkpoint inhibitors. Whether FLASH radiotherapy can replicate the significant advantages observed in preclinical studies in more complex scenarios (e.g., deep-seated tumors, combination with immunotherapy) remains to be confirmed by large-scale, randomized controlled Phase II/III clinical trials.

### 5.3 Theoretical benefits and practical limits of FLASH-RT in special populations

The formulation of radiotherapy regimens must fully account for patient age. Conventional radiotherapy (CONV-RT) in children can lead to severe late effects, including growth impairment, neurocognitive deficits, and secondary malignancies. In elderly patients, treatment is often compromised by poor tolerance, preventing the delivery of optimal doses. FLASH radiotherapy, with its superior normal tissue-sparing properties, offers a promising avenue to address these therapeutic dilemmas in both populations. The following sections will elaborate on the potential advantages and key scientific questions regarding FLASH-RT for pediatric and geriatric cancers.

In pediatric populations, FLASH radiotherapy exhibits distinctive clinical utility, leveraging its unique radiobiological properties to address the heightened radiosensitivity of developing tissues. Core advantages include the substantial mitigation of late-effect risks: it attenuates detrimental effects on growth plates, the central nervous system, and visceral organs, thereby reducing the incidence of severe long-term complications such as growth deformities, neurocognitive impairment, and secondary malignancies. Notably, the ultra-fast, sub-second treatment duration alleviates procedural anxiety and movement artifacts in children, decreases reliance on sedation and anesthesia, and enhances both treatment precision and efficiency. However, pediatric cohorts pose unique challenges: a pediatric-specific FLASH dose-response model is imperative to establish for precise delineation of therapeutic doses, long-term follow-up data spanning decades is lacking to validate safety throughout childhood development, and the FLASH beam must conform to small, complex target volumes while sparing critical organs, imposing stringent requirements on treatment planning systems (5).

In the elderly population, FLASH radiotherapy confers several clinically meaningful advantages that address age-related vulnerabilities. It augments tissue resilience by attenuating acute toxicities (e.g., radiation pneumonitis, dermatitis, enteritis) and the risk of late fibrosis, enabling even frail elderly individuals to tolerate radical radiotherapy regimens. Furthermore, it preserves immunological competence by mitigating radiotherapy-associated lymphopenia—a critical benefit for patients with age-related immune senescence—thereby sustaining anti-tumor immunity and overall treatment tolerance. The ultra-short treatment time also improves patient comfort and safety by minimizing time spent on the treatment table, making it particularly suitable for individuals with limited mobility. Conversely, elderly populations present distinct clinical challenges: interactions between FLASH radiotherapy and prevalent geriatric comorbidities (e.g., cardiovascular disease, diabetes) and their concomitant medications warrant rigorous investigation, a refined comprehensive geriatric assessment system—integrating physiological age, organ function, and nutritional status—is critical for individualized treatment stratification, and the complex tumor microenvironment in elderly patients necessitates further exploration of FLASH efficacy across diverse geriatric tumor models to optimize combination therapeutic strategies (156).

However, the potential benefits of FLASH radiotherapy in pediatric and elderly populations must be weighed against current technical realities. FLASH irradiation for the most common pediatric solid tumors—such as medulloblastoma, neuroblastoma, and Wilms tumor—requires intracranial or abdominal radiotherapy, which is not yet feasible with sufficient dosimetric quality. Until proton pencil beam scanning FLASH (PBS-FLASH) or very high-energy electron (VHEE) systems overcome their respective physical limitations, pediatric patients remain only conceptual beneficiaries of FLASH, not actual recipients.

### 5.4 Clinical radio-immunotherapy translation barriers

Despite the compelling preclinical rationale for the synergy between FLASH-RT and immunotherapy, the translation of this combination strategy into clinical practice faces unique and multifaceted barriers, stemming from the limitations of completed clinical trials, the absence of dedicated combination study data, and inherent design challenges for future FLASH-immune checkpoint inhibitors (ICIs) trials. These hurdles collectively hinder the validation of FLASH’s immunomodulatory potential in humans and the establishment of evidence-based clinical protocols.

Nearly all completed early-phase FLASH-RT clinical trials were designed with narrow, non-immunological primary objectives, with no integration of immune profiling into their study design or outcome assessment. The FAST-01 (10) and FAST-02 (156) trials, which evaluated FLASH-RT for painful bone metastases, focused exclusively on pain palliation and acute toxicity monitoring (e.g., skin injury, bone marrow damage), with no assessment of immune-related parameters such as peripheral lymphocyte subset composition, systemic cytokine/chemokine levels, or tumor-infiltrating lymphocyte (TIL) density and phenotype. Similarly, the IMPULSE trial, a Phase I dose-escalation study for cutaneous melanoma metastases, centers on defining the maximum tolerated dose (MTD) and evaluating acute skin reactions, with no collection of immunological correlates of treatment response or toxicity. The direct consequence of this endpoint design is a complete absence of human clinical data to validate, refute, or refine the immunomodulatory hypotheses of FLASH-RT derived from preclinical *in vitro* and animal models, creating a critical knowledge gap between bench and bedside for FLASH radio-immunotherapy.

To date, no clinical trial has reported clinical outcomes for the combination of FLASH-RT with any immune checkpoint inhibitor, representing the most significant translational bottleneck for this strategy. The only available evidence supporting FLASH-ICIs synergy comes from two preclinical studies: Eggold et al. (126) investigated abdominopelvic FLASH-RT combined with PD-1 inhibition in an ovarian cancer mouse model, while Shi et al. (20) evaluated X-ray FLASH combined with anti-PD-L1 therapy in a model of intestinal radiotoxicity and tumor control. These studies employed distinct tumor models, radiation sources (electron vs. X-ray FLASH), ICIs agents (anti-PD-1 vs. anti-PD-L1), and irradiation regimens, precluding direct comparison of their findings or generalization to human cancer. This dearth of clinical combination data means the safety, efficacy, and biological activity of FLASH-ICIs therapy in humans remain entirely uncharacterized.

The design of rigorous, informative clinical trials for FLASH-RT combined with ICIs is complicated by multiple unresolved questions regarding treatment delivery, patient selection, and endpoint assessment, all of which must be addressed to generate meaningful clinical evidence. Optimal sequencing of the two modalities remains an open question, as preclinical data suggest that administration of ICIs following FLASH-RT may maximize synergistic anti-tumor immunity by capitalizing on FLASH-induced immune activation and T cell infiltration into the tumor microenvironment, yet the optimal temporal window for ICIs administration post-FLASH remains undefined, with no consensus on whether a 24-hour, 72-hour, or 7-day interval yields the best balance of immune activation and toxicity mitigation. The immunologically optimal FLASH-RT dosing and fractionation strategy also lacks clinical definition, as most preclinical FLASH-immunotherapy studies utilized single doses of 15–30 Gy—a dosing approach that may not be clinically translatable for most solid tumors due to lingering concerns for off-target effects and normal tissue tolerance, even with FLASH’s well-documented tissue-sparing properties, and the relative merits of single high-dose irradiation versus hypofractionated delivery for immune modulation have not been established in human contexts.

Endpoint selection further complicates trial design, as traditional oncology trial endpoints such as local tumor control, progression-free survival, and overall survival may fail to capture the unique immune-mediated effects of FLASH-ICIs combination therapy, including abscopal responses or the development of durable immune memory that confers long-term protection against tumor recurrence. The incorporation of exploratory immune biomarkers is therefore essential to fully characterize the biological activity of this combination, including circulating tumor DNA (ctDNA) dynamics for real-time assessment of tumor burden and immune-mediated tumor cell killing, T cell receptor (TCR) repertoire sequencing to evaluate clonal T cell expansion and the development of tumor-specific immune responses, and multiplex immunohistochemistry (IHC) for comprehensive profiling of the tumor immune microenvironment post-treatment. Yet the integration of these specialized biomarkers adds significant complexity, financial cost, and logistical burden to trial design and implementation, requiring dedicated biorepository resources and specialized analytical expertise that may not be readily available across all clinical research centers.

Patient selection represents another critical design challenge, as the tumor types most likely to benefit from FLASH-ICIs combination therapy have not been identified. Trials must grapple with the decision to either prioritize “hot” tumors with pre-existing immune infiltration, such as melanoma and non-small cell lung cancer, which may be primed for FLASH-induced immune amplification and thus more likely to yield positive trial outcomes, or to attempt to convert immunologically “cold” tumors, such as pancreatic cancer and glioblastoma, with FLASH-RT prior to ICIs administration—an approach with far greater clinical potential for addressing currently untreatable malignancies but a correspondingly higher risk of treatment failure and trial underperformance. Compounding these challenges is the technical issue of dose rate consistency across the tumor target volume, particularly for proton FLASH-RT, a leading modality for clinical translation of the technology. Maintaining a consistent ultra-high dose rate (UHDR) throughout the spread-out Bragg peak (SOBP) remains a significant technical hurdle in proton FLASH delivery, and given that FLASH’s immunomodulatory effects are likely dose-rate dependent, inconsistent UHDR delivery across the tumor volume introduces confounding variables that may obscure the assessment of true FLASH-ICIs synergy and limit the interpretability of trial results.

The immunological rationale for combining FLASH-RT with ICIs—rooted in FLASH’s ability to preserve circulating immune cells, activate the tumor immune microenvironment, and downregulate immunosuppressive factors—is biologically compelling and supported by preclinical data. However, translational validation of this strategy in humans is completely lacking, due to the exclusion of immune endpoints in completed FLASH trials, the absence of published FLASH-ICIs combination study results, and substantial design challenges for future clinical investigations. Moving from preclinical promise to clinical proof-of-concept for FLASH radio-immunotherapy will require purpose-designed clinical trials that integrate immune monitoring and biomarker assessment from the outset. Additionally, collaborative efforts across research institutions, industry partners, and regulatory agencies are essential to standardize FLASH dosimetry, fractionation regimens, and immune endpoint reporting—ensuring that future trials generate comparable, actionable data to define the role of FLASH-ICIs combination therapy in cancer treatment.

### 5.5 Clinical translation bottleneck

The underlying biological mechanisms of the FLASH effect remain incompletely elucidated. Proposed hypotheses include radiolytic oxygen depletion (ROD), free radical recombination, enhanced DNA damage repair, and adaptive changes in mitochondrial function. These processes involve complex physicochemical interactions occurring within milliseconds post-irradiation, which significantly alter the tissue microenvironment and mitigate oxidative damage in normal tissues. However, none of the current mechanistic models can fully explain the FLASH effect independently, underscoring the necessity of integrating multi-scale and multi-mechanism perspectives.

Although FLASH-RT has demonstrated promising outcomes in preclinical studies, its clinical translation requires careful evaluation of tissue-specific radiotolerance. In CONV-RT, treatment efficacy is often constrained by dose limitations to adjacent critical organs; similarly, the clinical implementation of FLASH must account for the differential sensitivity of tissues to establish safe and effective irradiation protocols tailored to various anatomical sites.

Multiple studies have reported that the Dose Modifying Factor (DMF)—defined as the ratio of the dose required with CONV-RT to that with FLASH-RT to achieve the same biological effect—ranges from 1.1 to 1.8 across various normal tissues, indicating enhanced tissue tolerance under FLASH irradiation. For instance, a study on minipig skin demonstrated at least a 20% improvement in tolerance (DMF ≈ 1.2) (5); Ruan et al. reported a DMF of 1.1 in the intestine (158); and investigations in lung tissue revealed that while CONV-RT induced fibrosis at 17 Gray (Gy), FLASH-RT caused no complications below 30 Gy (DMF ≈ 1.8) (9). However, no protective effect was observed in mouse gonadal tissues, suggesting that the FLASH effect may exhibit tissue-specific dependence (155). This negative finding is not an isolated anomaly; it is a fundamental challenge to any unified theory of the FLASH effect. If oxygen depletion or radical recombination were the dominant mechanisms, they should operate in all metabolically active tissues. The gonadal exception therefore suggests that tissue-specific factors—such as baseline redox status, DNA repair capacity, mitochondrial dynamics, or immune privilege—can override the physicochemical effects of UHDR irradiation. Identifying why the gonad is refractory to FLASH may provide the key to deciphering the mechanism in protected tissues. Until this paradox is resolved, any claim of a “universal” FLASH mechanism remains premature.

Despite these encouraging findings, current research on tissue-specific radiotolerance remains limited, often constrained by narrow experimental dose ranges and methodological inconsistencies. Further systematic and standardized investigations are essential to establish precise organ-specific dose thresholds, which will be critical for the safe and effective clinical translation of FLASH-RT.

For successful clinical translation, parameters of FLASH-RT—including dose rate, total dose, and spatial dose distribution—must be meticulously optimized according to tumor characteristics, anatomical location, and individual patient factors. Monte Carlo (MC) simulation, a valuable tool for dose calculation in radiation therapy (RT), has been extensively investigated for the optimization of FLASH-RT, with various MC codes such as EGSnrc, DOSXYZnrc, and Geant4 being applied to simulate dose distributions and optimize FLASH-RT treatment plans (159).

Delivering ultra-high dose-rate FLASH-RT to deep, complex tumors is the biggest engineering challenge for clinical translation, as all current radiation modalities have unresolved physical limitations. VHEE offers sufficient penetration but poor distal dose fall-off and requires non-clinical accelerators only available at research facilities. Proton FLASH faces a fundamental conflict between pencil beam scanning and FLASH dose-rate requirements, and transmission proton FLASH sacrifices the Bragg peak with no way around this intrinsic constraint. Heavy-ion FLASH is even less developed, hampered by secondary particles, uncertain RBE, and extreme technical demands. Currently, no off-the-shelf clinical accelerator can deliver conformal FLASH-RT to deep tumors while sparing organs at risk; human FLASH studies are limited to superficial targets, meaning widespread clinical use requires fundamental breakthroughs in accelerator design.

Technological innovations, such as Stanford University’s PHASER system, represent early-stage engineering efforts; they have not yet been validated in clinical settings, and significant gaps remain. PHASER employs multiple high-energy electron beam sources equipped with rapid scanning capabilities and integrates Image-Guided Radiation Therapy (IGRT) for real-time tumor tracking. It dynamically adjusts beam direction and dose distribution to enhance targeting precision and reduce toxicity (160).

Similarly, optimization efforts for proton FLASH-RT are rapidly advancing. Recent studies conducted by Peking University and the New York Proton Center utilized genetic algorithms to optimize the delivery sequence of point-scanning proton beams, significantly enhancing the protection of organs at risk (OARs) while maintaining excellent dose uniformity within the target (161). Such innovations are critical for expanding the clinical applicability and safety profile of FLASH-RT. Through continued refinement of irradiation systems and personalized treatment planning, FLASH-RT may eventually become a clinically useful modality, but this will require resolution of the fundamental physical and dosimetric obstacles discussed above.

Given that FLASH-RT is characterized by ultra-high dose rates and sub-second irradiation times, accurate real-time dose monitoring during treatment is critically important (162). In CONV-RT, ionization chambers are widely employed for real-time monitoring of beam intensity and cumulative dose. However, under FLASH conditions, these detectors face significant limitations: the charge collection time of ionization chambers is substantially longer than the duration of FLASH pulses, resulting in delayed dose feedback and potentially insufficient time to interrupt irradiation in the event of an error. Furthermore, conventional ionization chambers cannot operate reliably under FLASH conditions due to ion recombination, signal saturation, and dose-rate dependence (163), while proposed correction factors are limited by device and energy specificity with a circular validation issue; all alternative dosimeters (Faraday cups, alanine/EPR, scintillators, film) have critical drawbacks, leaving no clinically viable real-time UHDR-compatible system. This means every FLASH treatment so far lacks the rigorous dosimetric verification required for standard radiotherapy, only being acceptable for compassionate-use research rather than routine care.

FLASH-RT has attracted growing attention from regulatory agencies and professional organizations worldwide. To date, however, neither the U.S. Food and Drug Administration (FDA) nor the European Medicines Agency (EMA) has approved its use in routine clinical practice. All current human applications are conducted within the framework of clinical trial protocols or compassionate use programs.

A major regulatory hurdle for FLASH therapy stems from its technical complexity. Since FLASH-RT requires treatment equipment to operate under extreme parameters—particularly involving ultra-high dose rates exceeding 40 Gy/s—it is generally classified as a major modification of existing systems, necessitating dedicated regulatory review and approval.

One of the most critical challenges in FLASH-RT is the absence of universally accepted definitions for key parameters such as dose-rate thresholds, dosimetry protocols, and quality assurance (QA) procedures. In response, international initiatives have advocated for the establishment of a centralized FLASH clinical research registry. Such a platform would standardize the reporting of essential metrics—including dose rate, fractionation schemes, toxicity profiles, and efficacy outcomes—thereby facilitating evidence-based regulatory evaluations and enabling robust cross-trial comparisons.

Notwithstanding these obstacles, progress toward regulatory approval of FLASH-RT is accelerating. Continued collaboration among research institutions, industry partners, and regulatory agencies remains paramount to ensure the safe and effective integration of this promising technology into clinical practice (164). Through concerted global efforts, FLASH-RT may soon transition from experimental investigations to mainstream cancer treatment.

## 6 Summary and outlook

With its disruptive irradiation mode characterized by an ultra-high dose rate (≥40 Gy/s), FLASH-RT effectively eradicates tumors while significantly reducing toxicity in normal tissues—a phenomenon known as the “FLASH effect”—thus inaugurating a new paradigm in cancer treatment. This review systematically summarizes its core advancements. FLASH exhibits unique physical properties that underpin the FLASH effect: millisecond-scale irradiation eliminates uncertainties caused by organ motion, and the ultra-high instantaneous dose rate alters the spatiotemporal distribution of free radicals (e.g., ·OH kinetics). Regarding the fundamental biological mechanisms, multiple hypotheses—including radiolytic oxygen depletion (ROD), free radical recombination, immune-mediated protection, preservation of DNA integrity, vascular normalization, and mitochondrial regulation—act synergistically to collectively account for the pronounced protective effects on normal tissues.

FLASH-RT may also offer distinct immunological properties that could render it particularly suitable for combination with immunotherapy, based on preliminary preclinical observations. By preserving circulating immune cells (reducing lymphocyte depletion rates to 5%–10%), activating the tumor immune microenvironment (enhancing CD8^+^T cell infiltration and promoting M1 macrophage polarization), and downregulating immunosuppressive factors (such as reducing TGF-β and PD-L1 expression), FLASH-RT acts synergistically with immunotherapies including immune checkpoint inhibitors (ICIs) and CAR-T cell therapy. This combination helps overcome the immunosuppressive limitations often associated with CONV-RT.

Preclinical animal studies have shown reproducible FLASH effects across multiple organs, yet inconsistent results and inter-study variability confirm these effects are neither universal nor fully understood. Early-phase clinical trials have verified the feasibility and acute safety of FLASH for superficial targets, but they lack efficacy data and do not resolve the technical barriers to treating deep-seated tumors, leaving a substantial gap between preclinical success and clinical benefit that will require years of cross-disciplinary research to close. Nevertheless, several challenges remain, including incomplete elucidation of its biological mechanisms, limitations in equipment and technical readiness, lack of standardized dosimetry protocols, and evolving regulatory frameworks.

Despite the significant preclinical potential of FLASH radiotherapy, the extensive evidence underpinning this review carries inherent assumptions and key limitations, necessitating prudence when extrapolating to clinical practice. Our understanding of FLASH’s biological mechanisms relies heavily on specific preclinical models (e.g., mice, cell lines), which differ from humans in immune function, tissue microenvironment, and DNA damage repair capacity, leaving the generalizability of conclusions unvalidated in more complex biological systems. Additionally, the precise measurement and standardization of ultra-high dose rates—the core of the FLASH effect—remain a major challenge, as inconsistent dose measurement equipment, methods, and definitions across laboratories introduce uncertainties in reported parameters, hindering the comparison, replication, and interpretation of findings. Compounding this, biological responses to FLASH radiotherapy vary markedly across species, tissue types, and individuals, complicating the establishment of universally optimal treatment parameters such as dose-rate thresholds. Most critically, clinical data on combining FLASH radiotherapy with immunotherapy are extremely scarce; the synergistic mechanisms discussed herein are largely based on theoretical speculation and limited preclinical observations, with FLASH still primarily in the conceptual and experimental stage and most studies remaining *in vitro* or preclinical. Even the few encouraging early clinical reports (e.g., for bone metastases or veterinary tumors) have not explored combinations with immunotherapies like immune checkpoint inhibitors, leaving the safety, efficacy, optimal sequencing, and long-term outcomes of such regimens entirely unknown and requiring rigorous future clinical trials. Acknowledging these limitations does not negate FLASH’s value but rather clarifies the boundaries of current knowledge, offering a balanced, transparent perspective to guide future research toward resolving these key uncertainties.

Overall, FLASH radiotherapy represents a potentially promising approach in radiation oncology, distinguished by its capacity to spare normal tissues while preserving antitumor efficacy. Its immunomodulatory properties offer a compelling, albeit still hypothetical, foundation for synergy with modern immunotherapies. However, this promise is currently balanced by substantial mechanistic uncertainties, technical hurdles, and a paucity of clinical evidence—particularly for combination regimens. Whether FLASH-RT will indeed influence treatment paradigms depends not on its theoretical advantages alone, but on the rigorous, interdisciplinary efforts needed to convert intriguing preclinical observations into reproducible, patient-benefiting therapies. The next decade will be critical in determining whether FLASH remains a niche investigational tool or evolves into a mainstream precision weapon against cancer.
